# [(Bromomethyl)phenyl]methyl-Conjugated Chalcone Derivatives as Potential Lung Cancer Inhibitors: Structure Modification, Molecular Docking, Molecular Dynamics and In Vitro Validation

**DOI:** 10.3390/ijms27146104

**Published:** 2026-07-08

**Authors:** Nathaporn Cheechana, Nopawit Khamto, Kraikrit Utama, Chawanakorn Kongsak, Puracheth Rithchumpon, Padchanee Sangthong, Puttinan Meepowpan

**Affiliations:** 1 Office of Research Administration, Chiang Mai University, Chiang Mai 50200, Thailand; nathaporn.c@cmu.ac.th (N.C.); kraikrit.utama@cmu.ac.th (K.U.); chawanakorn.kong@cmu.ac.th (C.K.); 2 Department of Chemistry, Faculty of Science, Chiang Mai University, 239 Huay Kaew Road, Chiang Mai 50200, Thailand; padchanee.sangthong@cmu.ac.th; 3 Department of Biochemistry, Faculty of Medical Science, Naresuan University, Phitsanulok 65000, Thailand; nopawitk@nu.ac.th; 4 Department of Chemistry, Faculty of Science, Khon Kaen University, Khon Kaen 40002, Thailand; purarit@kku.ac.th; 5 Center of Excellence in Materials Science and Technology, Chiang Mai University, 239 Huay Kaew Road, Chiang Mai 50200, Thailand; 6 Division of Biochemistry and Biochemical Innovation, Department of Chemistry, Faculty of Science, Chiang Mai University, Chiang Mai 50200, Thailand

**Keywords:** 2′,4′-dihydroxy-6′-methoxy-3′,5′-dimethylchalcone (DMC), [(bromomethyl)phenyl]methyl–chalcone, structure modification, lung cancer inhibitors, anti-lung cancer, molecular reactivity, molecular docking, molecular dynamics

## Abstract

Chalcones from medicinal plants are attractive starting points for anticancer lead discovery, and this study aimed to improve the anti-NSCLC potential of 2′,4′-dihydroxy-6′-methoxy-3′,5′-dimethylchalcone (DMC) from *Syzygium nervosum* through targeted structural modification and mechanistic computational analysis. Air-dried seeds were extracted with CH_2_Cl_2_, DMC was purified by silica gel chromatography and crystallization, and its structure was confirmed by HRMS, FTIR and NMR; 4′-*O*-derivatization with *meta*– or *para*–[(bromomethyl)phenyl]methyl groups afforded derivatives **2a** and **2b** (89–92% yield). Antiproliferative activity and cytotoxicity were evaluated by MTT assay in NSCLC cell lines (A549, NCI-H460) and normal lung fibroblasts (MRC-5) with osimertinib as a reference. Derivatives **2a** and **2b** showed enhanced activity against NCI-H460 (IC_50_ = 7.97 ± 0.62 µM and 7.91 ± 2.77 µM) while remaining weak against A549, and they were less cytotoxic to MRC-5 (IC_50_ ≥ 50 µM and 16.08 ± 0.45 µM) than osimertinib (IC_50_ = 3.43 ± 0.35 µM). DFT/MEP analyses, docking to wild-type EGFR (1M17; redocking RMSD 1.228 Å), and 500 ns × 3 replica MD simulations supported improved EGFR binding stability for **2a**/**2b**, with MM/GBSA indicating favorable binding free energies (−28.78 ± 6.11 and −21.99 ± 9.39 kcal/mol). Overall, 4′-*O*-functionalization of DMC generated lead derivatives with improved in vitro activity in NCI-H460 and computationally supported target–ligand stabilization.

## 1. Introduction

At present, many countries worldwide are confronted with air pollution problems, including those in Europe, Africa, North America, West Asia and the Asia–Pacific region. The World Health Organization (WHO) and United Nations Environment Programme (UNEP) have compiled global statistics on factors contributing to air pollution, such as residential activities, domestic and international transport, industrial activities, commerce, solvent use, windblown dust, combustion and agriculture [1]. Thailand is one of the countries that has long been affected by air pollution, particularly from January to June each year. During the winter-to-summer period (December–June), Thailand typically experiences relatively dry and stagnant atmospheric conditions. Consequently, increases in fine particulate matter (particulate matter with an aerodynamic diameter of 2.5 µm or less; PM_2.5_) have been observed in many areas of the country, especially in the northern region [2]. Owing to the topographical and climatic conditions, together with very low wind speeds and high atmospheric pressure, wildfires can readily occur in both Thailand and neighboring countries. As a result, the emitted particulate matter remains suspended in the atmosphere for longer than usual, leading to the accumulation of airborne pollutants. In addition, open biomass burning such as burning dried maize stalks, rice straw and leaf litter from deciduous dipterocarp and mixed deciduous forests also contributes to substantial emissions of fine particulate matter [3]. This pollution situation has widespread health impacts across all sexes and age groups, particularly among children and elderly people, whose immune function is generally lower than that of other age groups. Health effects associated with exposure to air pollution are linked to the development of non-communicable diseases (NCDs) in older adults. The mechanisms by which exposure to air pollutants, including PM_2.5_, contributes to disease involve oxidative stress, inflammatory responses and genotoxicity, which occur at the cellular level [4]. In addition, the mechanisms described above elicit distinct responses at the molecular level. Free radicals, organic chemicals and transition metals (e.g., manganese, copper, and iron) can generate reactive oxygen species (ROS) within cells. An increased intracellular production of ROS reduces the cell’s antioxidant capacity, thereby inducing oxidative stress. This impairment of the antioxidant defense system is associated with a reduction in nuclear factor erythroid 2–related factor 2 (Nrf2) [4,5]. Inflammation induced by exposure to PM_2.5_ is associated with the generation of reactive oxygen species (ROS) and the production of pro-inflammatory mediators implicated in the pathogenesis of various diseases, such as tumor necrosis factor-α (TNF-α), interleukin-1β (IL-1β), interleukin-6 (IL-6), interleukin-8 (IL-8, and monocyte chemoattractant protein-1 (MCP-1). In addition, PM_2.5_ can induce genotoxicity: the resulting ROS can damage genetic material at the DNA level, leading to DNA strand breaks and increased levels of 7-hydro-8-oxo-2′-deoxyguanosine (8-oxodG) and endonuclease III within cells, thereby exacerbating DNA damage [6]. These observations indicate that adverse health effects associated with exposure to PM_2.5_ are linked to the development of non-communicable diseases. Notably, lung cancer, an NCD, ranks among the leading causes of mortality worldwide, including in Thailand. According to lung cancer patient statistics reported by the National Cancer Institute, the northern region of Thailand has a higher number of lung cancer cases than other regions, which is consistent with increased levels of PM_2.5_. However, the mechanisms by which PM_2.5_ induces lung carcinogenesis remain unclear. Nevertheless, numerous studies have indicated that PM_2.5_ is a key contributor to lung cancer [7,8,9].

Lung cancer is characterized by the rapid, uncontrolled proliferation of abnormal cells, leading to the formation of tumor masses. It is often detected only when the tumour has grown substantially, increased in cell number and metastasised to other parts of the body. Lung cancer is broadly classified into two types according to tumour cell morphology: small cell lung cancer (SCLC) and non-small cell lung cancer (NSCLC). This distinction is critical for treatment planning. NSCLC is the most common type, accounting for approximately 90% of all lung cancer cases [10]. At present, lung cancer cannot be reliably cured, largely because most cases are diagnosed at advanced and metastatic stages. Consequently, clinicians must identify the most appropriate treatment strategies to prolong survival and improve patients’ quality of life. Key considerations in lung cancer management include tumour location, size and stage, as well as the patient’s physical and psychological status. Treatment strategies therefore depend on the stage at diagnosis. Current lung cancer treatment comprises five main modalities: surgery, radiotherapy, chemotherapy, immunotherapy and targeted therapy. Targeted therapy is an important approach for both early-stage and metastatic disease, with common targets including Kirsten rat sarcoma viral oncogene homolog (KRAS) and the epidermal growth factor receptor (EGFR). The KRAS oncogene regulates growth factor signaling involved in cell proliferation, growth, and survival. In lung cancer, the KRAS oncoprotein commonly harbors a G12C mutation in non-small cell lung cancer [11]. In addition, EGFR is a transmembrane receptor with tyrosine kinase activity that is implicated in regulating survival and metastasis in EGFR-mutant lung cancer cells. Therefore, KRAS and EGFR are key therapeutic targets for drug discovery and the development of treatments for lung cancer [12]. In previous studies, covalent inhibitors have been investigated for the development of KRAS and EGFR inhibitors. Currently available agents, including AMG510 (sotorasib), MRTX849 (adagrasib), osimertinib and erlotinib, have been approved by the Food and Drug Administration (FDA) as inhibitors of KRAS and EGFR, thereby suppressing survival and metastatic processes in lung cancer cells [11,13,14]. In addition to the examples mentioned above, a number of other agents have been synthesized and developed, and have received FDA approval for the treatment of lung cancer. For instance, afatinib (Gilotrif^TM^, Giotrif^®^) is an orally administered anticancer drug that functions as an irreversible inhibitor of the epidermal growth factor receptor family (ErbB tyrosine kinases). Afatinib modulates ErbB signalling by forming a highly stable covalent bond with the kinase domain of the EGFR at the active site, thereby permanently inhibiting tyrosine kinase autophosphorylation [15]. The advantages of targeted therapy in cancer treatment include a clear improvement in survival rates for certain cancers, tumour shrinkage in the targeted cancer cells, enhanced quality of life and the ability to control symptoms over a longer period. However, targeted therapies also have important limitations, including relatively high costs and restrictions on reimbursement. Their effectiveness is typically confined to patients with specific, confirmed driver mutations. Moreover, after a period of treatment, tumors may acquire new mutations or undergo other changes, resulting in resistance to the same targeted agent. In this study, we aim to develop lead compounds for targeted lung cancer therapy from medicinal plants containing naturally occurring phytochemicals. The goal is to identify candidates with anti-lung cancer activity comparable to that of current standard drugs, which could be further developed into therapeutic agents in the future.

Many researchers in Thailand and abroad have investigated bioactive compounds against various non-communicable diseases, including both synthetic chemicals and natural products. Thailand’s tropical geography contributes to high biodiversity, resulting in a wide variety of medicinal plants. These plants have been used for disease treatment since ancient times and have been passed down as household remedies or traditional medicine. Extracting compounds from medicinal plants and evaluating the biological properties of these extracts is an important and necessary approach for discovering new natural products from different plant species and for maximizing the use of domestic herbal resources. In this study, we are interested in extracting and structurally modifying bioactive compounds from a local plant, Ma-kiang (*Syzygium nervosum* A.Cunn. ex DC.), because it has been reported in Thai traditional herbal formulations to possess medicinal properties, contains key constituents with excellent bioactivity and enables the isolation of major bioactive compounds in relatively large quantities using relatively simple laboratory-scale chemical procedures. Ma-kiang (*Syzygium nervosum* A.Cunn. ex DC.) is a local plant in the family *Myrtaceae*, commonly found in Southeast Asian countries such as Thailand, Vietnam, China, Myanmar and Malaysia. The fresh fruits of Ma-kiang are valuable for Thailand’s food and beverage industry, for example, in the production of jam, fruit juice, tea, yogurt and wine. In addition, the leaves and young shoots of Ma-kiang are commonly used in traditional medicine to treat colds, inflammation, gastrointestinal disorders, and for antimicrobial purposes [16]. Phytochemical studies of *S. nervosum* have revealed numerous natural products, including terpenes, flavanones, flavones, flavonols, chalcones, anthocyanins and phloroglucinol derivatives. Moreover, these derivatives have been reported to exhibit anticancer activity and cytotoxic effects via multiple mechanisms [17,18]. 2′,4′-Dihydroxy-6′-methoxy-3′,5′-dimethylchalcone (DMC) is a major bioactive compound isolated from the seeds of *Syzygium nervosum*. It has been reported to exhibit multiple biological activities, including anticancer and antimicrobial effects, activation of AMP-activated protein kinase (AMPK), anti-influenza activity, and broader antiviral properties [19,20,21]. In particular, DMC has demonstrated moderate to excellent anticancer activity against a range of cancer cell lines, including human leukemia (K562) [22], pancreatic cancer (PANC-1) [23], cervical cancer (HeLa) [24], liver cancer (SMMC-7721) [24], breast cancer (MCF-7) [25] and lung cancer (A549) [26]. DMC contains hydroxyl groups at the C-2′ and C-4′ positions on aromatic ring A. In general, the 2′-OH group is less reactive than the 4′-OH group because it can form an intramolecular hydrogen bond with the adjacent carbonyl group. Structural modification can be carried out under mildly basic conditions *via* nucleophilic substitution reactions, allowing the preparation of a variety of DMC derivatives through organic synthetic methods. A structural optimization study of DMC by Nopawit Khamto and colleagues (2021) [27] modified DMC by introducing various functional groups at the 4′-OH position, including alkyl, benzoyl, sulfonyl, naphthoyl and allyl substituents. The resulting derivatives were then evaluated for cytotoxicity against six cancer cell lines, such as cholangiocarcinoma (KKU-M213), human squamous cell carcinoma (FaDu), human colorectal cancer (HT-29), breast cancer (MDA-MB-231), lung cancer (A549), and neuroblastoma (SH-SY5Y), and compared with a normal cell line (CL). The study found that 4′-*O* monosubstituted DMC derivatives exhibited high levels of cytotoxic activity. Overall, the results indicate that structural modification of DMC at the 4′-OH position is selective and can enhance its anticancer activity against cancer cells.

From the studies discussed above, DMC appears to play an important role in pharmacological activity and shows promising potential as an anti-lung cancer agent. Although there are still no clear clinical reports on treating lung cancer patients with extracts from this medicinal plant, well-designed and scientifically sound laboratory studies have been widely reported at the cellular level. Such findings can be used as a basis to guide and further develop research on new lead compounds for lung cancer therapy, which may later be advanced to animal studies and clinical evaluation. Accordingly, this research focuses on the identification of bioactive compounds and the structural modification of these compounds, with an emphasis on developing targeted inhibitors that selectively disrupt cellular signaling by inhibiting key receptor proteins implicated in lung cancer. In addition, we perform preliminary cell-based inhibitory assays and conduct molecular docking and molecular dynamics studies, together with electrostatic analyses and drug likeness evaluations, to support the potential effectiveness of the lead compounds investigated in this work.

## 2. Results

### 2.1. Extraction and Structure Modification of 2′,4′-Dihydroxy-6′-methoxy-3′,5′-dimethylchalcone (DMC) from Syzygium nervosum

Air-dried seeds of *S. nervosum* were pulverized and exhaustively extracted with CH_2_Cl_2_. The resulting crude extract was purified by silica gel column chromatography, eluting with a stepwise gradient of *n*-hexane/CH_2_Cl_2_ to afford fractions enriched in DMC. These DMC-containing fractions were subjected to further silica gel chromatography using an isocratic *n*-hexane/acetone mobile phase. Final purification was achieved by crystallization from a CH_2_Cl_2_/*n*-hexane solvent system, yielding orange needle crystals of DMC. The isolated compound, DMC (**1**), was obtained as orange needle crystals with a melting point of 124.3–126.0 °C. High-resolution ESI mass spectrometry (HRMS–ESI) exhibited a protonated molecular ion peak (M + H)^+^ at *m*/*z* 299.1283, corresponding to the molecular formula C_18_H_19_O_4_^+^ (calcd for *m*/*z* 299.1290). The FTIR spectrum displayed characteristic bands for an –OH stretching vibration and a conjugated ketone carbonyl at 3522–3125 and 1630 cm^−1^, respectively. The ^1^H-NMR spectrum showed resonances consistent with a monosubstituted aromatic ring at *δ* 7.38–7.43 and 7.63–7.66 ppm. Two olefinic protons corresponding to a trans *α*,*β*-unsaturated system were observed at *δ* 7.84 and 7.99 ppm, each exhibiting large vicinal coupling constants (*J* = 15.6–15.7 Hz). A methoxy group appeared as a singlet at *δ* 3.66 ppm, and benzylic methyl signals were detected at *δ* 2.14 and 2.16 ppm. In addition, two hydroxyl proton signals at *δ* 5.44 and 13.61 ppm were assigned to a free hydroxyl group and a chelated (intramolecularly hydrogen-bonded) hydroxyl group, respectively.

**Scheme 1:**
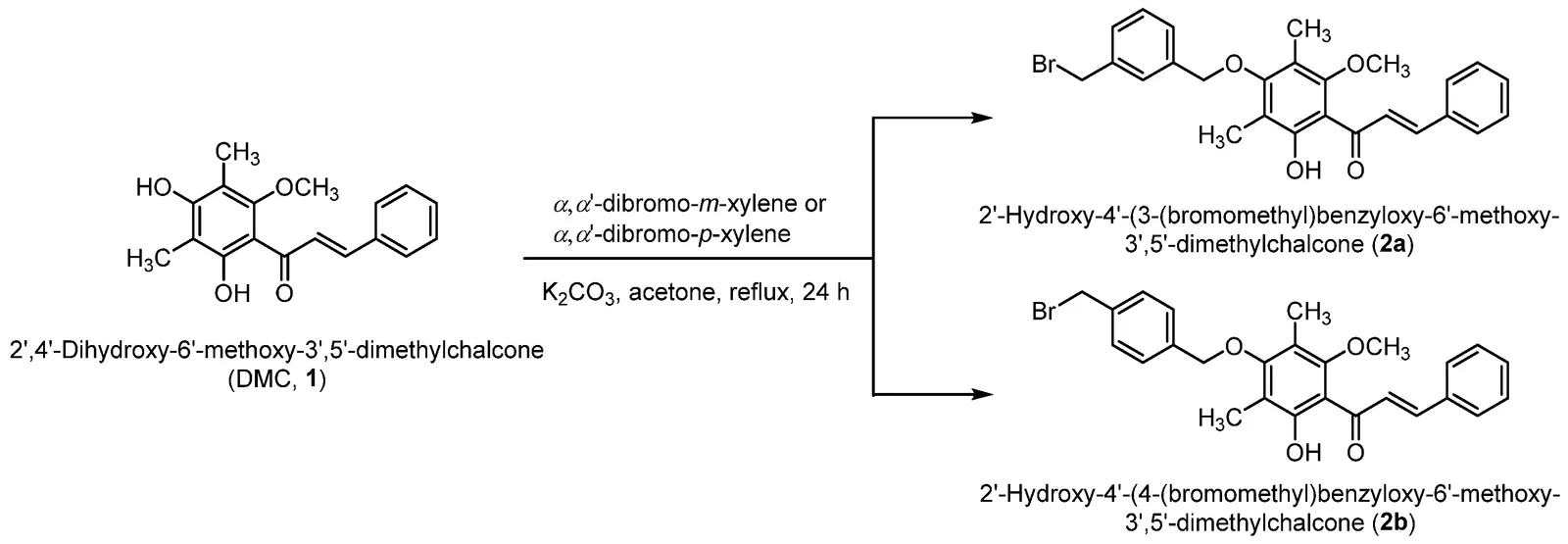
Synthesis of [(bromomethyl)phenyl]methyl-conjugated DMC derivatives **2a** and **2b**.

Chemical optimization of DMC was directed mainly toward derivatization at the 4′-hydroxyl group, since this position is highly reactive and has been previously implicated in improving biological activity in related analogs. We initiated this study by *O*-acylating DMC with a series of [(bromomethyl)phenyl]methyl groups substituted at the *meta*- and *para*-positions, as outlined in Scheme 1. DMC derivatives **2a** and **2b** were synthesized through a standard organic reaction of DMC with *α*,*α*′-dibromo-*m*-xylene or *α*,*α*′-dibromo-*p*-xylene, respectively, using K_2_CO_3_ as a base in acetone. The reaction was conducted under reflux for 24 h. Following purification, the products were obtained as orange viscous liquids in high yields of 89% and 92%, respectively. Representative FTIR spectra of 2′-hydroxy-4′-(3-(bromomethyl)benzyloxy)-6′-methoxy-3′,5′-dimethylchalcone (**2a**) and 2′-hydroxy-4′-(4-(bromomethyl)benzyloxy)-6′-methoxy-3′,5′-dimethylchalcone (**2b**) showed the appearance of a new intense band at 1685 cm^−1^, attributed to the C–O stretching vibration of the substituent introduced at the 4′-*O* position, confirming *O*-functionalization. Consistent with this transformation, the ^1^H-NMR spectra no longer displayed the 4′-OH proton signal of the parent DMC. Instead, new methylene resonances corresponding to the appended [(bromomethyl)phenyl]methyl fragment were observed at *δ* 4.53 ppm (–CH_2_–Br) and *δ* 4.58 ppm (–CH_2_–O–). In agreement, the ^13^C-NMR spectra exhibited new signals for these methylene carbons at *δ* 33.3 and 74.2 ppm, further supporting successful formation of the 4′-*O*-substituted derivatives.

### 2.2. Antiproliferative Activity of [(Bromomethyl)phenyl]methyl–DMC Derivatives (***2a*** and ***2b***) on Non-Small Cell Lung Cancer Cell Line (A549 and NCI-H460) and Normal Human Fetal Lung Fibroblast Cell Lines (MRC-5)

The antiproliferative effects against lung cancer cells and cytotoxicity toward normal cells of the structurally optimized, natural product-derived compounds were evaluated using an MTT assay in non-small cell lung cancer cell lines (A549 and NCI-H460) and a normal human lung fibroblast cell line (MRC-5) (Table 1). Activities were benchmarked against osimertinib, a clinically used targeted therapy for lung cancer. Compounds were considered promising when the selectivity index (SI) > 2, indicating preferential inhibition of cancer cells over normal cells in vitro. The parent compound DMC (**1**) showed moderate cytotoxic activity against both cancer cell lines (IC_50_ = 14.74 ± 2.57 µM for A549 and 20.80 ± 2.02 µM for NCI-H460). However, it also exhibited comparable activity toward normal MRC-5 cells (IC_50_ = 11.45 ± 0.71 µM), resulting in low selectivity indices. The results demonstrated that DMC derivatives **2a** and **2b** showed enhanced activity against the NCI-H460 cell line, with IC_50_ values of 7.97 ± 0.62 µM and 7.91 ± 2.77 µM, respectively, outperforming osimertinib (IC_50_ = 14.10 ± 0.35 µM). In contrast, both derivatives exhibited only weak activity against A549 cells. Overall, these findings indicate that structural modification of the parent natural product DMC (**1**) improved anti-NSCLC potency, particularly toward NCI-H460 cells. Cytotoxicity testing further indicated that **2a** and **2b** were comparatively less toxic to MRC-5 normal fibroblasts, with IC_50_ values of ≥50 µM and 16.08 ± 0.45 µM, respectively, whereas osimertinib was substantially more cytotoxic (IC_50_ = 3.43 ± 0.35 µM). Taken together, the two derivatives were identified as lead candidates combining potent anti-NSCLC activity with reduced toxicity toward normal lung cells. These leads were then advanced to computational analyses including MEP mapping, PES calculations, molecular docking and molecular dynamics simulations to explore structure activity relationships and ligand–EGFR binding behavior in support of future optimization efforts.

### 2.3. Molecular Reactivity Analyses

Properties derived from the HOMO–LUMO energies are widely used to evaluate molecular reactivity and chemical stability. These descriptors are especially valuable for interpreting and predicting behavior in organic synthesis (e.g., electrophilic/nucleophilic tendencies) and in biomedical research (e.g., interactions relevant to binding and biological activity) [28]. In this study, density functional theory (DFT) was used to optimize the molecular structure and obtain reliable bond length and bond angle parameters. Geometry optimization of the synthesized compound was carried out using the B3LYP functional with the 6–31G(d,p) basis set, and the optimized geometrical parameters (bond lengths and bond angles) were extracted from the resulting minimum-energy structure. HOMO–LUMO orbital characteristics were examined for both the ground (most stable) state and the first excited (higher-energy) state using the DFT method with the 6–31G(d,p) basis set. To account for solvent effects, all calculations were carried out with the SMD implicit solvation model, employing different solvent environments, specifically DMSO and water. The influence of solvation on the electronic structure was evaluated by comparing the HOMO and LUMO energies and the resulting HOMO–LUMO energy gap (ΔE) across the solvent conditions; these results are presented in Table 2. From the DFT-B3LYP/6–31G(d,p) frontier orbital energies, additional global reactivity descriptors were derived to describe the molecule’s tendency to donate or accept electrons. These included the highest occupied molecular orbital energy (E_HOMO_) and lowest unoccupied molecular orbital energy (E_LUMO_), as well as chemical hardness (η) and chemical softness (s), which reflect resistance or susceptibility to charge transfer. Furthermore, the electron-donating (ω^−^) and electron-accepting (ω^+^) were calculated to quantify donating/accepting ability under each solvation condition [29]. All computed descriptor values are summarized in Table 3.

A comparison of the HOMO–LUMO energy gap (ΔE) between unmodified DMC (**1**) and the functionalized derivatives **2a** and **2b** shows that DMC (**1**) has a smaller (narrower) ΔE, whereas **2a** and **2b** exhibit larger (wider) ΔE values. In frontier-orbital terms, a wider ΔE generally corresponds to a molecule that is electronically more stable (harder), with lower polarizability and a reduced tendency to undergo intramolecular charge redistribution in response to its environment. Therefore, the broader ΔE values observed for **2a** and **2b** are consistent with derivatives that are more electronically rigid and less “soft,” which can translate into more predictable, geometry-driven binding. From a protein–ligand interaction perspective, this implies that **2a** and **2b** may bind in a way that depends more on: steric/shape complementarity (good fit within the pocket), hydrophobic effects (favorable burial of nonpolar surface) and directional hydrogen bonding (specific donor–acceptor alignment), rather than being dominated by strong electronic reorganization or charge-transfer character. In other words, their binding can be viewed as being stabilized primarily by classical noncovalent contacts and conformational compatibility, supporting more consistent residence within the binding site. In contrast, DMC (**1**) shows a narrower ΔE in both implicit solvent models (water and DMSO), which indicates an electronically softer and more polarizable framework. A smaller gap reflects an enhanced ability to redistribute electron density when exposed to changing electrostatic environments such as moving from bulk solvent into a heterogeneous protein pocket. As a result, DMC’s binding behavior is more likely to include interaction components where electronic effects are important, for example: charge-transfer contributions (donor–acceptor orbital mixing), aromatic π-driven interactions such as π–π stacking and electrostatics involving aromatic systems, including π–cation and π–anion contacts. Overall, the ΔE trend suggests that **2a** and **2b** are better described as electronically stable (harder) binders whose affinity may arise largely from fit and localized H-bonding, whereas DMC (**1**) is a softer, more electronically responsive ligand whose binding may be more sensitive to the local electrostatic and π-interaction landscape of the target site. For osimertinib, the HOMO–LUMO gap (ΔE) was larger in the aqueous model than for the other compounds (3.8668 eV), but became smaller in the DMSO model (3.8395 eV). This solvent-dependent shift suggests that in DMSO, the solvent commonly used to prepare compounds for cell-culture assays, the osimertinib adopts a relatively “softer” and more polarizable electronic structure. As a result, its interactions in the protein binding pocket may rely more heavily on electrostatic-field effects and electron-density redistribution, which can translate into less consistent binding stability compared with the more electronically stable DMC derivatives **2a** and **2b**. In this context, the increased electronic softness in DMSO provides a plausible explanation for why osimertinib shows lower inhibitory potency than **2a** and **2b** in the cell-based experiments, since its binding may be more sensitive to the surrounding electrostatic environment and less dominated by rigid, shape-complementary contacts.

Regarding the chemical hardness (η) and chemical softness (s) descriptors, the results indicate that DMC derivatives **2a** and **2b** are chemically harder than unmodified DMC (**1**) and osimertinib. This pattern matches the HOMO–LUMO gap (ΔE) trend and supports the conclusion that **2a** and **2b** are more electronically stable, less polarizable and more resistant to electron-density redistribution. Consequently, once positioned in the protein binding pocket, these derivatives are expected to maintain their electronic structure more consistently, which can contribute to more stable binding overall. In contrast, DMC (**1**) and osimertinib show greater chemical softness, indicating that they can more readily reorganize electron density in response to the surrounding environment. This higher polarizability implies that their binding may be more strongly influenced by local electrostatic fields and electronically driven interaction components, leading to interactions that can be more environment-dependent than those of the harder derivatives. Considering the electrodonating (ω^−^) and electroaccepting (ω^+^) descriptors, the calculations show that all compounds have larger ω^−^ than ω^+^, with this difference being most pronounced for the lead derivatives **2a** and **2b**. This trend indicates that these ligands are dominated by an electron-donating character and therefore have a stronger propensity to donate electron density than to accept it. As a result, they would be expected to preferentially interact with positively polarized/positively charged regions of the protein environment. Moreover, the presence of aromatic rings in the lead structures provides π-electron density that can facilitate π-driven interactions, including π–π stacking with aromatic amino acid residues, and can also contribute to donor–acceptor behavior through the aromatic π-systems, potentially strengthening binding within the pocket. Overall, these results outline predicted trends in the molecular reactivity of the lead ligands toward the target protein. The principal binding interactions and binding free energies of the lead compounds with wild-type EGFR are discussed in detail in the molecular docking and molecular dynamics sections.

### 2.4. Molecular Electrostatic Potential Surface Analysis

The molecular electrostatic potential (MEP) and electrostatic potential surfaces are informative descriptors for representing the overall charge distribution of a molecule and for visualizing how electrostatic character varies across its structure. Because the potential at each point around a molecule reflects the combined effect of nuclei and electron density, MEP maps clearly distinguish electron-rich regions (more negative potential) from electron-poor regions (more positive potential). This spatial distribution is directly relevant to molecular recognition, since it helps explain where and how a molecule is likely to interact with another species through electrostatic attractions, hydrogen bonding and other noncovalent forces. For this reason, MEP is widely employed as a qualitative reactivity map in organic and medicinal chemistry. Regions with more negative potential are typically associated with sites that can attract electrophiles or act as hydrogen-bond acceptors, while regions with more positive potential are more susceptible to nucleophilic attack or can behave as hydrogen-bond donors (depending on functional groups). In practice, identifying these high- and low-potential zones supports the prediction of preferred binding orientations, the likely positions of key intermolecular contacts and the relative contribution of electrostatic complementarity when a ligand approaches a protein binding pocket.

MEP surfaces and the corresponding electrostatic potential contour maps were computed for the studied compounds using the DFT/B3LYP method with the 6–31G(d,p) basis set, applying DMSO and water as solvent models. The calculated MEP surfaces and contour plots for the title compound are provided in Table 4. Electrostatic potential values on the MEP surface are illustrated using a color gradient. In this scheme, red denotes regions with the most negative electrostatic potential, white denotes regions with the most positive electrostatic potential and blue represents areas where the potential is close to zero. Accordingly, the potential increases along the color scale in the order red < green < blue < pink < white [30]. The optimized structures obtained in both the water and DMSO solvent models show that the regions of most negative electrostatic potential are mainly concentrated around electronegative functionalities. For DMC (**1**) and its derivatives **2a** and **2b**, the strongest negative potential is predominantly localized near the oxygen-containing groups, particularly the hydroxyl (–OH) and carbonyl (–C=O) moieties, and it is also pronounced around the Br atom. This distribution indicates that these sites carry relatively higher electron density and are therefore more likely to participate in electrostatically favorable contacts, such as hydrogen-bond acceptance or interactions with positively polarized residues in the binding environment. In contrast, osimertinib displays negative potential spread over a wider portion of its aromatic ring system and the carbonyl (–C=O) group. This broader delocalization suggests a more extended π-electron contribution to the electrostatic potential across the aromatic framework. Overall, the red-colored regions on the MEP surfaces correspond to areas of higher electron density compared with the rest of the molecular surface, highlighting the most probable regions for electrophilic approach and electrostatic complementarity during intermolecular interactions. The regions of positive electrostatic potential (white areas) were mainly concentrated around hydrogen atoms, including those of methyl and methylene groups as well as aromatic protons. Overall, the MEP maps indicated a clear separation of electrostatic character: the negative potential sites are primarily associated with oxygen-containing functionalities, whereas the positive potential sites are largely distributed around hydrogen atoms. Identifying these complementary regions helps predict where the compounds are most likely to participate in noncovalent interactions, particularly electrostatic contacts and hydrogen-bonding interactions, with surrounding molecules such as residues in the protein binding pocket.

### 2.5. Molecular Docking

**Figure 1:**
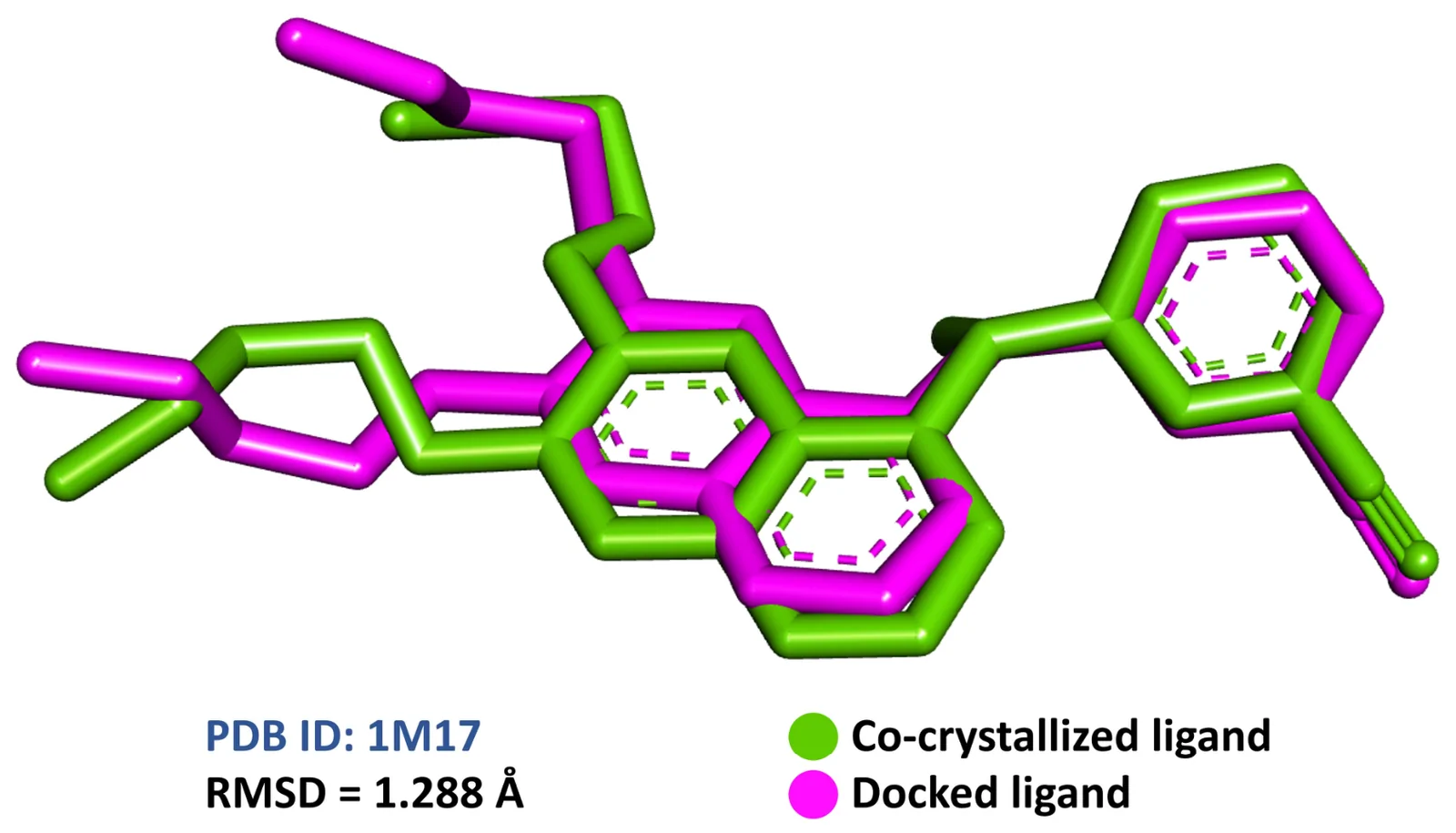
Overlay of the redocked ligand (pink) with the crystallographic reference ligand (green) in the wild-type EGFR (PDB ID: 1M17), showing an RMSD of 1.288 Å.

In this study, wild-type EGFR (PDB ID: 1M17) was chosen as the target for both molecular docking and molecular dynamics simulations. This selection was based on the outcomes of the inhibition assays performed using osimertinib as the positive control. Osimertinib is a clinically approved third-generation targeted therapy for lung cancer and is known for its high specificity toward EGFR. Notably, the lead compounds in this work demonstrated stronger inhibitory activity against the NCI-H460 lung cancer cell line than the positive control. Accordingly, the wild-type EGFR structure was used to enable a broader and more comprehensive evaluation of ligand–protein binding behavior and interaction stability. The RMSD obtained from the redocking validation in this study was 1.228 Å, indicating that the selected receptor model and docking protocol were suitable and reliable for docking calculations. The superimposed pose of the redocked ligand and the corresponding crystallographic reference ligand is shown in Figure 1, demonstrating good agreement between the predicted and experimental binding conformations.

**Figure 2:**
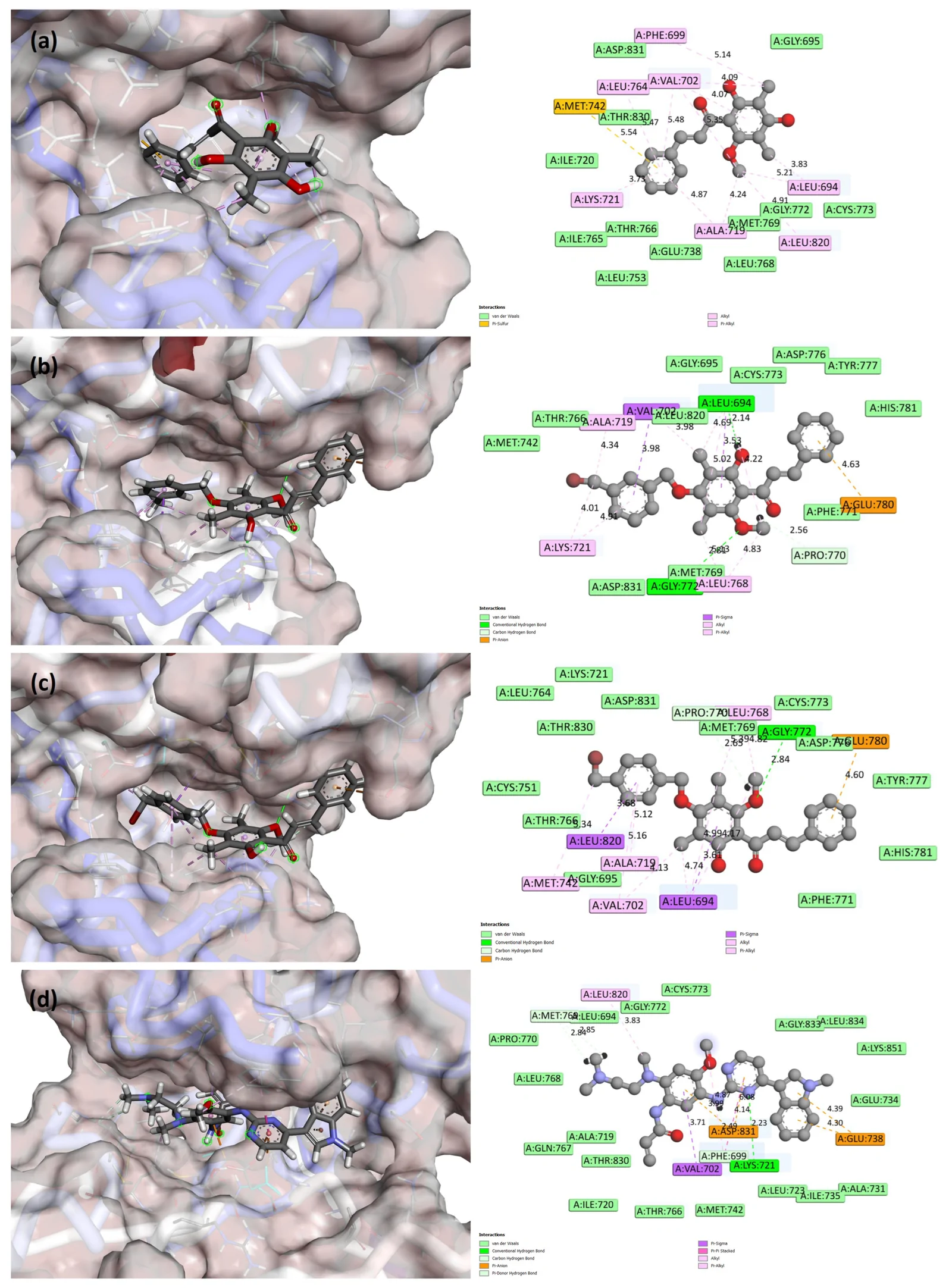
Three-dimensional (3D) and two-dimensional (2D) molecular docking interaction profiles of (**a**) DMC (**1**), (**b**) **2a** derivative, (**c**) **2b** derivative and (**d**) osimertinib in the binding site of wild-type EGFR (PDB ID: 1M17).

Based on the molecular docking analysis against wild-type EGFR (PDB ID: 1M17), the structurally optimized DMC derivatives **2a** and **2b** showed clearly enhanced predicted binding free energies (ΔG) relative to the parent compound DMC (**1**). In particular, **2a** and **2b** produced ΔG values of −8.6 and −8.8 kcal mol^−1^, respectively, compared with −7.7 kcal mol^−1^ for DMC (**1**), indicating stronger predicted interactions with the EGFR binding site after structural modification. Notably, the reference inhibitor osimertinib exhibited a comparable docking score (ΔG = −8.5 kcal mol^−1^), placing it in the same predicted affinity range as the lead derivatives. Overall, the binding-energy trend can be summarized as **2b** ≈ **2a** ≈ osimertinib > DMC (**1**). At 298 K, the calculated binding constants (Ka) suggest that derivatives **2a** and **2b** provide the strongest predicted affinity toward the target protein and are closely comparable in binding performance. Among all ligands, **2b** yields the highest Ka (2.84 × 10^6^ M^−1^), while **2a** follows with a slightly lower value (2.02 × 10^6^ M^−1^). The relatively small separation between these constants indicates that both derivatives possess similarly high binding propensity under the modeled conditions, with **2b** showing only a modest advantage. Osimertinib exhibits a moderately high Ka (1.72 × 10^6^ M^−1^), indicating favorable predicted binding but reduced association relative to **2a** and **2b**. In contrast, the parent compound DMC (**1**) displays the lowest Ka (4.43 × 10^5^ M^−1^), implying the weakest predicted interaction with the protein among the four candidates. Overall, the predicted binding trend is **2b** ≥ **2a** > osimertinib >> DMC (**1**). This pattern supports the conclusion that the designed DMC derivatives possess more favorable predicted binding free energies than the parent scaffold and suggests that the applied structural modifications improved receptor–ligand complementarity within wild-type EGFR. These docking results are also consistent with the experimentally observed stronger inhibitory activity of **2a** and **2b** against the NCI-H460 lung cancer cell line, supporting their potential as improved wild-type EGFR targeting candidates for further investigation. Based on the interaction analysis between wild-type EGFR (PDB ID: 1M17) and DMC (**1**), derivatives **2a** and **2b**, and osimertinib (Table 5). The DMC (**1**) was found to interact with the binding pocket mainly through van der Waals contacts and π–alkyl interactions. In addition, a π–sulfur interaction was observed between aromatic Ring B of DMC (**1**) and the sulfur atom of Met742. For derivatives **2a** and **2b**, the observed binding interactions were in good agreement with the molecular electrostatic potential (MEP) surface analysis. Notably, compound **2a** established two conventional hydrogen bonds: (i) the hydrogen atom of the 2′-OH group formed a hydrogen bond with the carbonyl oxygen of Leu694, and (ii) the methoxy oxygen substituent (present in both **2a** and **2b**) acted as a hydrogen-bond acceptor toward the hydrogen atom of the –NH group of Gly772. These hydrogen-bonding sites are consistent with the MEP characteristics of the ligands, where the methoxy oxygen and carbonyl oxygen correspond to electron-rich regions (red), while the 2′-OH hydrogen corresponds to an electron-poor region (blue). This complementary charge distribution rationalizes the enhanced hydrogen-bonding potential of the derivatives within the wild-type EGFR binding pocket. Beyond hydrogen bonding, additional electrostatic and hydrophobic contacts were observed. In particular, π–anion interactions were identified between aromatic ring B of both derivatives and an oxygen atom of Glu780. Furthermore, π–sigma interactions were detected between the aromatic ring of the [(bromomethyl)phenyl]methyl substituent and the methyl groups of Leu694, Val702 and Leu820, respectively, further stabilizing the ligand–EGFR complexes. For osimertinib, one conventional hydrogen bond was identified between the nitrogen atom of the aromatic ring (hydrogen-bond acceptor) and the hydrogen atom of the –NH_3_ group of Lys721 (hydrogen-bond donor). Notably, this site on osimertinib corresponds to a high-electron-density region (red) in the MEP map, supporting its strong hydrogen-bonding capability and consistent with the MEP analysis. In addition, π–anion interactions were observed between the aromatic ring of osimertinib and the oxygen atoms of Glu738 and Asp831. Moreover, a π–sigma interaction was detected between the aromatic system of osimertinib and the methyl group of Val702, and π–π stacking was observed with the aromatic ring of Phe699, further contributing to stabilization of the ligand within the wild-type EGFR binding pocket. Beyond qualitative classification of the interaction types, quantitative reporting of hydrogen-bond distances constitutes a critical component for substantiating the binding interaction analysis between the derivatives and the protein [31]. In addition, hydrogen-bond distances at each interaction site between the derivatives and wild-type EGFR were evaluated, as summarized in Table 6. The analysis indicated that derivative **2a** formed two hydrogen bonds with Leu694 and Gly772, with bond lengths of 2.14 and 2.81 Å, respectively. Notably, the hydrogen atom of the 2′-OH group formed a hydrogen bond with the carbonyl oxygen of Leu694, exhibiting a shorter distance and thus suggesting a stronger interaction. This interaction may be an important contributor to stabilizing the binding of derivative **2a**. Regarding derivative **2b** and osimertinib, derivative **2b** exhibited a single hydrogen-bond interaction with Gly772, with an H-bond distance of 2.84 Å, indicative of moderate interaction strength. Likewise, osimertinib formed one hydrogen bond with Lys721, with a bond length of 2.23 Å, suggesting a relatively strong interaction. Nevertheless, this distance remained longer than that observed for derivative **2a**. The 3D and 2D interaction diagrams are shown in Figure 2. The molecular docking results were in good agreement with the in vitro antiproliferative activity against lung cancer cells. To further elucidate the stability and dynamic behavior of the wild-type EGFR–ligand complexes formed by the most potent inhibitors, **2a** and **2b**, we subsequently performed molecular dynamics simulations.

### 2.6. Molecular Dynamics

**Figure 3:**
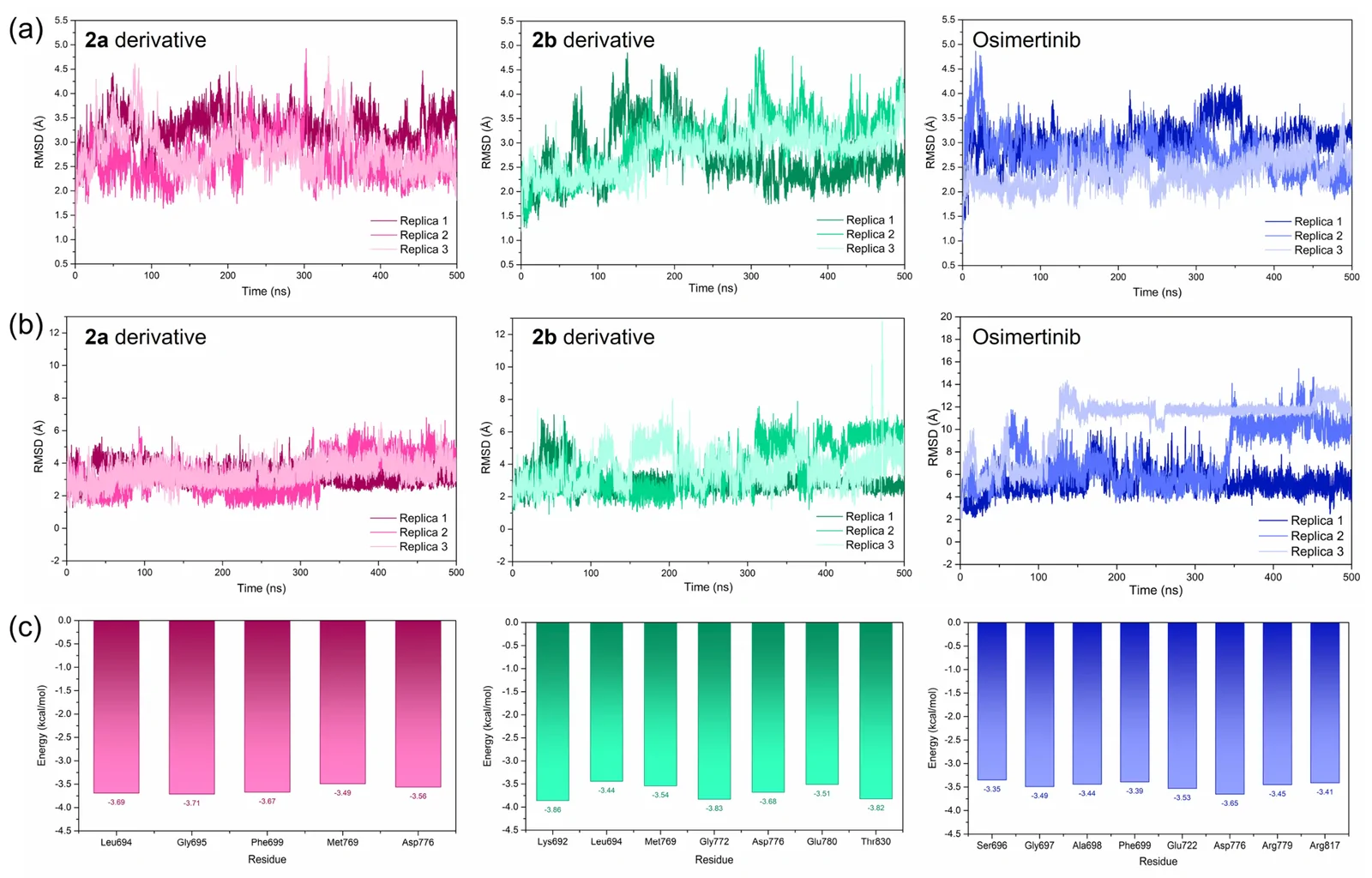
Molecular dynamics simulations (500 ns; three independent replicas) of the wild-type EGFR (PDB ID: 1M17) in complex with the potent anti-lung cancer agents DMC derivatives **2a**, **2b** and osimertinib. (**a**) Protein backbone root-mean-square deviation. (**b**) Ligand root-mean-square deviation. (**c**) Hydrogen-bonding interactions identified from the per-residue energy decomposition analysis.

All-atom classical molecular dynamics simulations were performed to investigate the stability and dynamic behavior of wild-type EGFR (PDB ID: 1M17) in complex with compounds **2a**, **2b** and osimertinib. For each system, three independent replicas were simulated for 500 ns. The time evolution of each complex was assessed by calculating the root-mean-square deviation (RMSD) of the protein backbone and the bound ligand, as shown in Figure 3a,b. Figure 3a presents the time evolution of the protein backbone root-mean-square deviation for wild-type EGFR in complex with each ligand. The pink trace corresponds to the EGFR–**2a** complex, the green trace to the EGFR–**2b** complex and the blue trace to the EGFR–osimertinib complex, respectively. Across three independent replicas, the backbone RMSD trajectories showed an initial adjustment phase followed by convergence, with all complexes reaching an apparent equilibrium after 200 ns. After this point, RMSD values fluctuated within a relatively narrow range without evidence of progressive drift, indicating that the protein–ligand complexes remained structurally stable for the remainder of the simulation time. Across the three ligand-bound EGFR systems, the protein backbone RMSD values were highly comparable, with RMSD values of 2.90 ± 0.32 Å (EGFR–**2a**), 2.85 ± 0.14 Å (EGFR–**2b**), and 2.71 ± 0.33 Å (EGFR–osimertinib). The similar Cα RMSD magnitudes across systems indicate that the overall protein conformation was well equilibrated under all ligand-bound conditions. Notably, no abnormal RMSD excursions or sustained drift were observed, suggesting the absence of large-scale structural perturbations during the simulations. This structural stability supports the reliability of subsequent analyses focused on ligand retention and binding mode stability throughout the trajectories.

Ligand RMSD profiles are shown in Figure 3b. The RMSD ligand values were 3.31 ± 0.06 Å for **2a**, 3.44 ± 0.46 Å for **2b** and 7.73 ± 2.64 Å for osimertinib. Derivative **2a** exhibited the most stable behavior, as reflected by its minimal variability, indicating that **2a** retained a consistent binding orientation within the pocket throughout the simulation and thus showed high binding-pose stability. Derivative **2b** produced a comparable average RMSD, likewise supporting a stable binding mode, although with greater fluctuation than **2a**. In contrast, osimertinib showed a markedly higher ligand RMSD and substantially larger dispersion, consistent with increased positional or conformational mobility within the binding site. This pattern suggests that osimertinib underwent pronounced rearrangements relative to its initial pose, potentially involving displacement toward the pocket periphery and intermittent partial disengagement from key binding interactions. Overall, the ligand RMSD analysis indicates that derivatives **2a** and **2b** maintain more persistent binding poses than osimertinib under the simulated conditions.

Based on the hydrogen-bond interaction analysis of the final 5000 trajectory frames (Figure 3c), derivative **2a** formed persistent hydrogen bonds with Leu694, Gly695, Phe699, Met769 and Asp766. The associated interaction energies were narrowly distributed (−3.49 to −3.71 kcal/mol), indicating consistent hydrogen-bond strengths and supporting a stable binding pose within the active site over the analyzed simulation window. For derivative **2b**, hydrogen-bonding interactions were detected with a larger set of residues than observed for 2a. Nevertheless, several key residues were common to both complexes, including Lys692, Leu694, Met769, Gly772, Asp776, Glu780 and Thr830. The associated interaction energies spanned −3.44 to −3.86 kcal/mol, consistent with stable hydrogen-bond contributions within the binding site. In the osimertinib-bound system, hydrogen-bond analysis revealed a partially overlapping interaction pattern with those of derivatives **2a** and **2b**, but with distinct residue preferences. These differences are likely driven by the greater positional and conformational instability of osimertinib within the binding pocket, in agreement with its wider ligand RMSD fluctuations. As a result, osimertinib engaged additional hydrogen-bond partners, including Ser696, Gly697, Ala698, Phe699, Glu722, Asp776, Arg779 and Arg817. The corresponding interaction energies were in the range of −3.35 to −3.65 kcal/mol, indicating overall weaker hydrogen-bond contributions relative to derivatives **2a** and **2b**. For subsequent binding free energy calculations and protein–ligand interaction analyses, equilibration of each system was evaluated over the simulation trajectory using RMSD profiles of both the protein backbone and the ligand. Replicates demonstrating the highest stability, which is defined as minimal ligand displacement within the most conformationally consistent protein binding site, were selected for detailed assessment: replica 3 for the **2a** complex, replica 1 for the **2b** complex and replica 1 for the osimertinib complex.

Relative binding free energies were estimated using the MM/GBSA method, as summarized in Table 7. In the gas-phase component of the MM/GBSA analysis, the van der Waals (vdW) term was more favorable than the electrostatic term for all three ligands. This trend suggests that complex formation is driven predominantly by nonpolar/dispersion contacts rather than by long-range Coulombic interactions. Accordingly, vdW interactions appear to play a central role in stabilizing the binding of derivatives **2a** and **2b** and osimertinib within the EGFR pocket, consistent with a recognition mechanism that relies strongly on shape complementarity and hydrophobic packing against the surrounding residues. The two potent DMC derivatives displayed favorable MM/GBSA binding free energies of −28.78 ± 6.11 kcal/mol for **2a** and −21.99 ± 9.39 kcal/mol for **2b**. Importantly, derivative **2a** showed the most favorable binding among the studied ligands, with a binding free energy that was more negative than that of osimertinib (−12.19 ± 6.36 kcal/mol), suggesting stronger predicted affinity and a more stable bound state under the simulated conditions. In contrast, derivative **2b** exhibited a less favorable binding free energy than **2a**, indicating comparatively weaker predicted binding. In addition, the lowest binding energy observed for osimertinib implies greater fluctuation in its binding energetics across the analyzed frames, consistent with its higher ligand mobility inferred from the RMSD analysis. Electrostatic interactions contributed to complex stabilization but were not the dominant driving force. Among the three ligands, osimertinib exhibited the most favorable electrostatic term, followed by derivative **2b** and then derivative **2a**. This trend suggests that osimertinib relies more strongly on polar/charge-assisted contacts, whereas the derivatives, particularly **2a**, achieve high affinity primarily through other components (e.g., stronger van der Waals packing), despite having weaker electrostatic contributions. The polar solvation term showed a similar trend to the electrostatic interactions. In other words, ligands with stronger electrostatic interactions also experienced a larger (more unfavorable) polar desolvation penalty. Based on the polar solvation energies, the magnitude followed the order: osimertinib > derivative **2b** > derivative **2a**. This indicates that osimertinib incurred the greatest polar solvation penalty upon binding, whereas derivatives **2a** and **2b** have the smallest penalty, which may partly offset its weaker electrostatic contribution and support its overall favorable binding free energy. Osimertinib exhibited the largest entropic penalty (19.74 ± 1.76), in agreement with its least favorable ΔG_bind_. Although **2b** displayed the smallest −TΔS term (7.83 ± 7.42), its binding remained weaker than that of **2a**, indicating that the observed affinity ranking is predominantly dictated by enthalpic and solvation contributions that outweigh the entropic advantage of **2b**. In contrast, **2a** yielded the most favorable ΔG_bind_ despite a moderate entropic cost (13.18 ± 5.09), suggesting the presence of stronger compensatory interactions that stabilize the bound state. The MM/GBSA-derived ΔG_bind_ values aligned well with the antiproliferative results. Derivatives **2a** and **2b**, which produced the strongest growth inhibition against NCI-H460 lung cancer cells, also showed more favorable predicted binding free energies than osimertinib, indicating higher estimated binding affinity in this system. This consistency between computational energetics and cellular activity supports the conclusion that rational structural optimization of the DMC scaffold to generate **2a** and **2b** substantially improves the biological potential of the parent framework. Therefore, these derivatives represent promising lead candidates and merit further investigation, including expanded pharmacokinetic evaluation, pharmacological network or target pathway analyses and in vivo efficacy studies in relevant animal models.

## 3. Discussion

This study combined natural product isolation, rational structural modification and multi-level biological/computational evaluation to identify improved chalcone-derived leads against non-small cell lung cancer. Overall, the results support the working hypothesis that functionalization of the reactive 4′–hydroxyl position of 2′,4′-dihydroxy-6′-methoxy-3′,5′-dimethylchalcone (DMC) can enhance anticancer potential by improving receptor–ligand complementarity and stabilizing productive binding within the wild-type EGFR pocket, which in turn is consistent with stronger antiproliferative activity in a susceptible NSCLC model.

DMC (**1**) was isolated from *Syzygium nervosum* seeds and structurally confirmed by HRMS and spectroscopic analyses. Key diagnostic features, including the trans *α*,*β*-unsaturated system (large coupling constants), oxygenated substituents and a chelated hydroxyl signal, are consistent with a chalcone framework capable of forming both hydrogen-bonding and π-driven interactions. Such features are often considered advantageous in medicinal chemistry because they offer defined “interaction handles” while retaining a planar aromatic-enone scaffold that can engage hydrophobic and aromatic residues in protein pockets. Building on this rationale, the present work prioritized derivatization at 4′-OH, a chemically accessible site that has been implicated in modulating biological activity in related phenolic scaffolds. The successful 4′-*O*-functionalization to afford derivatives **2a** and **2b** in high yields (89–92%) is important beyond synthetic feasibility: it establishes a practical route for systematic structure–activity relationship (SAR) exploration around a natural product core. In vitro testing showed that **2a** and **2b** markedly improved antiproliferative potency against NCI-H460 cells (IC_50_ ~8 μM), outperforming osimertinib in this model, while remaining weak against A549. This cell-line dependence is a common feature in anticancer screening and is often interpreted as reflecting differences in oncogenic drivers, signaling dependencies, transporter activity, metabolic inactivation or apoptotic competence across cell types. In this context, the selective sensitivity of NCI-H460 suggests that the optimized DMC derivatives may engage a vulnerability more prominent in this cellular background. Importantly, both derivatives, especially **2a**, showed reduced cytotoxicity toward normal lung fibroblasts (MRC-5) relative to osimertinib. From a translational perspective, early signals of preferential inhibition of cancer cells over normal cells (SI criterion) are valuable because they imply a wider in vitro therapeutic window and provide justification for deeper mechanistic and optimization studies rather than relying solely on potency.

The DFT-based reactivity descriptors (HOMO–LUMO gap, hardness/softness, ω^−^/ω^+^) and MEP surface analyses offer a coherent framework for interpreting why 4′-*O*-substitution improved performance. The broader HOMO–LUMO gaps and increased hardness of **2a**/**2b** indicate more electronically stable, less polarizable ligands compared with DMC (**1**). In medicinal chemistry terms, “harder” ligands often display binding that depends less on extensive electronic reorganization and more on steric complementarity, hydrophobic packing and directional hydrogen bonding features that can support reproducible binding poses. Conversely, the narrower gap and greater softness of DMC suggest stronger sensitivity to heterogeneous electrostatic environments within a protein pocket, potentially increasing dependence on context-specific π/electrostatic contributions. Consistently, MEP surfaces localized the most negative electrostatic potentials mainly at heteroatom-rich regions (*O*-containing groups) and around Br for DMC derivatives, highlighting likely sites for favorable electrostatic contacts and hydrogen-bond acceptance. In contrast, osimertinib displayed broader delocalization of negative potential across aromatic systems, implying a different balance of π-contributions and polar contacts. Together, these results support the interpretation that DMC derivatives were engineered toward ligands with clearer electrostatic and a binding mode more dominated by geometry-driven, localized noncovalent interactions.

Molecular docking against wild-type EGFR (PDB ID: 1M17) provided direct evidence that the derivatives improve predicted affinity relative to the parent scaffold (ΔG = −8.6 to −8.8 kcal/mol vs. −7.7 kcal/mol for DMC). The redocking RMSD (~1.23 Å) supports the reliability of the docking protocol and lends confidence to comparative interpretation across ligands. Notably, **2a**/**2b** ranked similarly to osimertinib in docking score, aligning with the observed strong activity in NCI-H460. Interaction fingerprints also shifted in a manner consistent with rational optimization. While DMC relied largely on van der Waals and π–alkyl contacts (with a π–sulfur contact to Met742), **2a**/**2b** formed more structured networks combining conventional hydrogen bonds (e.g., involving Leu694 and Gly772), π–anion interactions (e.g., with Glu780) and additional π–sigma/hydrophobic contacts contributed by the introduced aromatic substituent. This pattern matches the MEP predicted charge complementarity and supports a mechanistic explanation that derivatization increased both interaction diversity and pocket fit two features commonly associated with improved binding and, potentially, higher cellular potency when the target is relevant in the cellular context.

Long-timescale MD (3 replicas × 500 ns) indicated that the wild-type EGFR backbone remained stable across all ligand-bound systems after equilibration (~200 ns), suggesting that differences in ligand behavior are not artifacts of global protein destabilization. The ligand RMSD analysis, however, clearly distinguished the derivatives from osimertinib: **2a** showed the most stable bound orientation (low variability) and **2b** remained generally stable but fluctuated more, while osimertinib exhibited substantially higher ligand RMSD and dispersion, consistent with increased mobility and intermittent weakening of key interactions. Hydrogen-bond analyses further support this interpretation: **2a** maintained persistent H-bonding with a focused set of residues and narrow interaction energy distributions, consistent with a stable pose. Osimertinib showed broader residue engagement but weaker overall contributions and greater variability, in agreement with its mobility. Importantly, MM/GBSA estimates reinforced the stability hierarchy: **2a** showed the most favorable binding free energy (more negative than osimertinib), whereas osimertinib was less favorable than **2a** and **2b**. Decomposition trends indicated that van der Waals interactions dominate binding for all ligands, suggesting that hydrophobic packing and shape complementarity are key drivers in this pocket. Osimertinib exhibited stronger electrostatic terms, but also incurred a larger polar desolvation penalty, highlighting a classic binding tradeoff where strong polar contacts can be offset by unfavorable desolvation. In contrast, **2a** achieved strong net binding with comparatively smaller polar solvation penalties, offering a plausible physicochemical basis for its favorable MM/GBSA profile and stable MD behavior. Taken together, the in silico results provide a consistent mechanistic narrative: 4′-*O*-substitution yields ligands that maintain stable poses through favorable dispersion-driven packing and well-positioned localized hydrogen bonds, which aligns with the improved antiproliferative activity observed in NCI-H460. While docking/MD cannot alone prove wild-type EGFR is the sole cellular target, the agreement between predicted binding stability/energetics and cell-based potency strengthens the argument that wild-type EGFR engagement is at least a plausible contributor to the observed biological effects.

These findings place with **2a** and **2b**, particularly **2a**, as promising leads that combine improved anti-NSCLC potency with lower toxicity to normal lung fibroblasts and computationally supported binding stability in wild-type EGFR. Future work should (i) validate target engagement in cells (e.g., pathway readouts downstream of EGFR and binding/competition assays), (ii) broaden biological profiling across additional NSCLC models to clarify the A549/H460 response gap and identify predictive biomarkers of sensitivity, and (iii) expand SAR around the 4′-*O*-substituent to balance van der Waals packing, hydrogen-bond geometry and desolvation penalties. In parallel, early developability studies such as metabolic stability, permeability and in vivo tolerability/efficacy will be important to determine whether the improved binding stability translates into favorable pharmacological behavior beyond the cell assay context.

## 4. Materials and Methods

### 4.1. General Experimental Procedure

Melting points were determined using a Gallenkamp Electrothermal apparatus (Synoptics Ltd., Beacon House, Nuffield Road, Cambridge, UK). FTIR spectra were recorded on a Bruker TENSOR 27 spectrometer (Bruker Optik GmbH, Ettlingen, Germany), and absorption maxima (*ν*_max_) are reported in cm^−1^. High-resolution mass spectra (HRMS; *m*/*z*) were obtained by ESI-QTOF-MS/MS on an Agilent QTOF 6540 UHD mass spectrometer (Agilent Technologies, Singapore). ^1^H-NMR and ^13^C-NMR spectra were acquired on a Bruker NEO^TM^ 500 spectrometer (Bruker Corporation, Ettlingen, Germany) to aid structure elucidation and signal assignment. Chemical shifts (*δ*) are reported in ppm relative to TMS or referenced to residual solvent resonances (CHCl_3_ in CDCl_3_: *δ*_H_ 7.26 ppm; *δ*_C_ 77.16 ppm). NMR data are presented as *δ*, multiplicity and coupling constants (*J*, Hz). Multiplicities are denoted as *s* (singlet), *d* (doublet), *t* (triplet) and *m* (multiplet); broad signals are indicated by *br*. Column chromatography was performed on SiliaFlash P60 silica gel (40–63 µm, 230–400 mesh; Silicycle, Quebec City, Canada). Reaction progress was monitored by TLC on Merck silica gel PF_254_ aluminum plates. All reactions were conducted in oven-dried glassware under a nitrogen atmosphere using anhydrous solvents, which were dried and distilled prior to use according to standard procedures.

### 4.2. Extraction, Isolation of DMC from Syzygium nervosum Seeds (Ma-Kiang) and Structural Characterization by ^1^H-NMR, ^13^C-NM, and HRMS-ESI

Building on a previous report describing the isolation and structural characterization of 2′,4′-dihydroxy-6′-methoxy-3′,5′-dimethylchalcone (DMC) from *Syzygium nervosum* seeds [27], DMC was reisolated following an analogous procedure to provide starting material for chemical modification. Powdered, air-dried seeds (10 kg) were exhaustively macerated with dichloromethane (CH_2_Cl_2_, 40 L) at room temperature (3 extractions, 5 days each). After filtration, the combined CH_2_Cl_2_ extracts were concentrated under reduced pressure to yield a dark-green viscous crude extract (398.3 g). This material was subjected to silica gel column chromatography using a gradient of *n*-hexane/CH_2_Cl_2_ (100:0 to 80:20 *v*/*v*). DMC-enriched fractions were combined and further purified by silica gel column chromatography eluting with *n*-hexane/acetone (90:10 *v*/*v*), affording an orange precipitate. Final purification by recrystallization from CH_2_Cl_2_/*n*-hexane gave DMC (5.4428 g) as orange, needle-shaped crystals. The structure was verified by comparison of the spectroscopic data with published reference values.

2′,4′-Dihydroxy-6′-methoxy-3′,5′-dimethylchalcone (DMC) (**1**). Orange needle-shaped crystals; mp 124.3–126.0 °C (CH_2_Cl_2_/*n*-hexane); *ν*_max_ (ATR) 3522–3125 (O–H), 3089–3001 (C–H), 2948 (–CH_3_) 1630 (C=O), 1601 (C=C), 1454–1421 (C–H aliphatic bending), 1167–1112 (C–O–C), 691 (C–H out of plane aromatic) cm^−1^; *δ*_H_ (500 MHz, CDCl_3_) 2.13 (*s*, 3H, –CH_3_), 2.16 (*s*, 3H, –CH_3_), 3.66 (*s*, 3H, –O–CH_3_), 5.46 (*s*, 1H, –OH), 7.37–7.45 (*m*, 3H, aromatic proton), 7.61–7.68 (*m*, 2H, aromatic proton), 7.84 (*d*, *J* = 15.7 Hz, 1H, methine proton), 7.99 (*d*, *J* = 15.7 Hz, 1H, methine proton), 13.61 (*s*, 1H, –OH); *δ*_C_ (125 MHz, CDCl_3_) 7.6, 8.3, 62.4, 106.6, 108.9, 109.1, 126.8, 128.5, 129.0, 130.3, 135.4, 143.0, 158.9, 159.3, 162.1, 193.4; HRMS (ESI) calcd for C_18_H_18_O_4_H^+^ (M + H)^+^: *m*/*z* 299.1283, found 299.1290.

### 4.3. General Procedure for the Synthesis of [(Bromomethyl)phenyl]methyl-Conjugated DMC Derivatives (***2a*** and ***2b***)

In a 50 mL round-bottom flask, DMC (50 mg, 0.17 mmol) was combined with *α*,*α*′-dibromo-*m*-xylene (for **2a**) or *α*,*α*′-dibromo-*p*-xylene (for **2b**) (442.38 mg, 1.70 mmol) (C_8_H_8_Br_2_, Acros Organics BV (Thermo Fisher Scientific), Janssen Pharmaceuticalaan 3a, Geel, 2440, Belgium, 96%) and K_2_CO_3_ (23.16 mg, 0.17 mmol). Acetone (20 mL) was added, and the resulting mixture was heated at reflux with continuous stirring for 24 h. The course of the reaction was followed by TLC using CH_2_Cl_2_/*n*-hexane as the developing solvent. After TLC indicated complete consumption of the starting material, the reaction mixture was neutralized with 1 M HCl to approximately pH 6–7. The suspension was vacuum-filtered to remove inorganic salts, and the filtrate was transferred to a separatory funnel and partitioned between CH_2_Cl_2_ and H_2_O. The organic layer was separated, dried over anhydrous Na_2_SO_4_, filtered and concentrated under reduced pressure to give a crude residue. Purification was carried out by silica gel column chromatography, eluting with a CH_2_Cl_2_/*n*-hexane gradient. Fractions were collected in 20 mL portions, analyzed by TLC and those containing the desired product were combined and concentrated in vacuo to afford the corresponding product, which was then subjected to spectroscopic characterization. DMC derivatives **2a** and **2b** were isolated as orange viscous liquids, with 89% and 92% yield, respectively.

2′-Hydroxy-4′-(3-(bromomethyl)benzyloxy-6′-methoxy-3′,5′-dimethylchalcone (**2a**). Orange viscous liquid; *ν*_max_ (ATR) 3064 (C–H aromatic), 2925 (–CH_2_), 2858 (–CH_3_), 1685 (C=O), 1588–1562 (C=C), 1344 (C–H aliphatic bending), 1147 (C–O–C), 1111 (C–O–C), 614–577 (C–Br) cm^−1^; *δ*_H_ (500 MHz, CDCl_3_) 2.18 ppm (*d*, *J* = 7.3 Hz, 6H, –CH_3_), 3.67 ppm (*s*, 3H, –OCH_3_), 4.53 ppm (*s*, 2H, –CH_2_–), 4.85 ppm (*s*, 2H, –CH_2_–), 7.37–7.46 ppm (*m*, 6H, aromatic protons), 7.49–7.52 ppm (*m*, 1H, aromatic protons), 7.63–7.70 ppm (*m*, 2H, aromatic protons), 7.88s ppm (*d*, *J* = 15.7 Hz, 1H, methine proton), 7.99 ppm (*d*, *J* = 15.7 Hz, 1H, methine proton) and 13.10 ppm (*s*, 1H, 2′–OH); *δ*_C_ (125 MHz, CDCl_3_) 9.1, 9.3, 13.9, 18.6, 36.0, 62.7, 113.3, 115.3, 115.8, 126.5, 128.7, 129.1, 130.6, 135.2, 143.9, 154.3, 158.3, 160.9, 170.8, 194.5; HRMS (ESI) calcd for C_26_H_25_BrO_4_H^+^ (M + H)^+^: *m*/*z* 481.1014, found 481.0980.

2′-Hydroxy-4′-(4-(bromomethyl)benzyloxy-6′-methoxy-3′,5′-dimethylchalcone (**2b**). Orange viscous liquid; *ν*_max_ (ATR) 3061 (C–H aromatic), 2925 (–CH_2_), 2858 (–CH_3_), 1697 (C=O), 1587–1562 (C=C), 1345 (C–H aliphatic bending), 1147 (C–O–C), 1111 (C–O–C), 610–577 (C–Br) cm^−1^; *δ*_H_ (500 MHz, CDCl_3_) 2.17 ppm (*d*, *J* = 6.7 Hz, 6H, –CH_3_), 3.67 ppm (*s*, 3H, –OCH_3_), 4.53 ppm (*s*, 2H, –CH_2_–), 4.84 ppm (*s*, 2H, –CH_2_–), 7.39–7.49 ppm (*m*, 7H, aromatic protons), 7.63–7.70 ppm (*m*, 2H, aromatic protons), 7.88 ppm (*d*, *J* = 15.7 Hz, 1H, methine proton), 7.98 ppm (*d*, *J* = 15.7 Hz, 1H, methine proton) and 13.10 ppm (*s*, 1H, 2′–OH); *δ*_C_ (125 MHz, CDCl_3_) 9.1, 9.3, 13.9, 18.6, 36.0, 62.7, 113.3, 115.3, 115.8, 126.5, 128.7, 129.1, 130.6, 135.2, 143.9, 154.3, 158.3, 160.9, 170.8, 194.5; HRMS (ESI) calcd for C_26_H_25_BrO_4_H^+^ (M + H)^+^: *m*/*z* 481.1014, found 481.1010.

### 4.4. Cell Culture

The human lung carcinoma cell lines A549 (CCL-185^TM^) and NCI-H460 (HTB-177^TM^) and the normal human lung fibroblast cell line MRC-5 (CCL-171^TM^) were obtained from the American Type Culture Collection (ATCC). Cells were grown in Dulbecco’s Modified Eagle Medium (DMEM) supplemented with fetal bovine serum (10% *v*/*v*) and penicillin–streptomycin (1%; stock 10,000 U/mL) and maintained at 37 °C in a humidified atmosphere containing 5% CO_2_.

### 4.5. Cytotoxicity and Antiproliferative Lung Cancer Activities of DMC (***1***), ***2a*** and ***2b*** Derivatives by MTT Assay

MRC-5, A549 and NCI-H460 cells were seeded in 96-well plates at 5 × 10^3^ cells/mL and incubated for 24 h at 37 °C in a humidified atmosphere containing 5% CO_2_. The parent compound and semi-synthetic derivatives were then added at concentrations of 0–50 µM, and the cells were incubated for an additional 48 h. The culture medium was subsequently aspirated and the cells were washed with PBS (pH 7.4). MTT solution (5 mg/mL) was added to each well, and the plates were incubated for 4 h at 37 °C under 5% CO_2_. Formazan formation was stopped by dissolving the crystals in DMSO and absorbance was measured at 550 nm with a reference wavelength of 620 nm using a SpectraMax i3x multi-mode microplate reader (Molecular Devices, San Jose, CA, USA). Percentage cell viability and IC_50_ values were calculated by nonlinear regression (curve fitting) in GraphPad Prism version 8.0.

### 4.6. Computational Studies

#### 4.6.1. Molecular Electrostatic and Potential Energy Surface Calculations

All quantum chemical calculations for the title compound were performed with Gaussian 16 (Revision C.02) and molecular visualization/figure preparation was carried out using GaussView 6.0 [32]. Conformational sampling *via* potential energy surface (PES) scans, frontier molecular orbital (HOMO–LUMO) analysis and molecular electrostatic potential (MEP) mapping were conducted at the RMP2–FC/6–31G(d,p) level, employing the frozen-core approximation. Geometry optimizations were subsequently carried out within the DFT framework using the B3LYP functional in conjunction with the 6–31G(d,p) basis set. Vibrational frequency calculations were performed at the B3LYP/6–31G(d,p) level to obtain harmonic wavenumbers (cm^−1^) along with related spectroscopic and mechanical parameters, including IR intensities (KM mol^−1^), Raman scattering activities (Å^4^ amu^−1^), depolarization ratios (for plane and unpolarized incident light), reduced masses (amu), force constants (mDyne·Å^−1^) and normal-mode displacement vectors (normal coordinates). The computed B3LYP/6–31G(d,p) harmonic vibrational data were corrected to compensate for the systematic overestimation inherent to the harmonic approximation and the chosen level of theory.

#### 4.6.2. Molecular Docking and Molecular Dynamics

The three-dimensional structure of wild-type EGFR (PDB ID: 1M17) [33] was retrieved from the Protein Data Bank. The co-crystallized ligand present in the crystal structure was removed prior to docking. Molecular docking was performed using AutoDock Vina [34]. Protein preparation was carried out in BIOVIA Discovery Studio Visualizer 2020 by deleting crystallographic water molecules, ligands and ions. Protonation states of ionizable residues were assigned at pH 7.0 using the PROPKA server (PDB2PQR; https://server.poissonboltzmann.org/pdb2pqr) (accessed on 3 June 2026) [35]. The [(bromomethyl)phenyl]methyl-conjugated chalcone derivatives (**2a** and **2b**) and the reference inhibitor osimertinib were built in GaussView 6.0 and geometry optimized in Gaussian 16 at the DFT/B3LYP/6–31G(d,p) level. Ligands were converted to PDBQT format using AutoDockTools v1.5.6 with Gasteiger charges assigned. The docking grid was defined based on the binding site of the co-crystallized erlotinib, with the grid center set to x = 21.697 Å, y = 0.303 Å and z = 52.093 Å, and box dimensions of 42 × 14 × 20 Å to encompass the binding pocket. Osimertinib was used as the control compound for the in silico study. Docking outputs were analyzed and visualized using BIOVIA Discovery Studio Visualizer 2020. Regression fitting of the binding isotherms yielded the equilibrium association constant (Ka) and the enthalpy change upon binding (ΔH). The Gibbs free energy of binding (ΔG) was subsequently computed from Ka at temperature T according to ΔG = −RTln(Ka), where R is the universal gas constant and T is the absolute temperature (K) [36].

Molecular dynamics (MD) simulations of the docked **2a**–EGFR and **2b**–EGFR complexes were performed using the pmemd.CUDA engine in AMBER 16 [37] under periodic boundary conditions. Simulations were conducted under an isothermal–isobaric (NPT) ensemble and repeated in triplicate. The protein was parameterized using the ff14SB force field, while ligand parameters were assigned using the General AMBER Force Field 2 (GAFF2). Ligand partial charges were derived using a standard RESP protocol. Briefly, the three-dimensional structures of the **2a** and **2b** derivatives were first geometry-optimized at the DFT/B3LYP/6–31G(d,p) level using Gaussian 16. Electrostatic potential (ESP) calculations were then performed and used to generate restrained electrostatic potential (RESP) charges within the AMBER 16 suite. The protein–ligand complexes were embedded in a cubic simulation box, leaving a minimum distance of 12 Å between the protein surface and the box boundary. Each system was solvated with explicit TIP3P water, and Na^+^/Cl^−^ counterions were added to achieve electroneutrality and a physiological ionic strength of 0.15 M. AMBER parameter/topology files were subsequently converted to GROMACS-compatible topologies using ParmEd (Python script) (part of AmberTools distributed with Amber/2022-cpeGNU-CUDA-11.7). After system setup, a two-step energy minimization protocol was applied. First, the structures were minimized using the steepest-descent algorithm until the maximum force fell below 10 kJ·mol^−1^·nm^−1^. This was followed by an additional refinement using the conjugate-gradient method to further relax any remaining unfavorable contacts. The minimized complexes were then equilibrated with positional restraints in two consecutive phases. The first phase employed an NVT ensemble for 1000 ps to stabilize the temperature. The second phase used an NPT ensemble for a further 1000 ps to allow density and box dimensions to converge. During both equilibration stages, the temperature was controlled at 310.15 K and the pressure was maintained at 1 bar. Ligand coordinates were kept under positional restraints during the equilibration (NVT and NPT) stages. For all simulation steps, temperature and pressure were regulated using a modified Berendsen thermostat for thermal coupling and a Parrinello–Rahman barostat for pressure coupling. Short-range van der Waals interactions were treated using a 12 Å cutoff. Long-range electrostatic interactions were handled with the particle-mesh Ewald (PME) approach, employing a real-space cutoff of 12 Å. For production dynamics, three independent MD runs of 500 ns each were carried out using a 2 fs integration time step and coordinates were written to the trajectory file every 10 ps. All-atom RMSD was computed for each system to evaluate overall structural stability during the simulations. Binding free energy calculations were performed using the gmx_MMPBSA package [38]. A total of 5000 snapshots extracted from the equilibrated portion of the trajectories were used for analysis. The binding free energy (ΔG_bind_) was estimated using the MM-GBSA approach. For all MM-GBSA calculations, the internal dielectric constant (*intdiel*) and external dielectric constant (*extdiel*) were set to 1 and 78.5, respectively. The salt concentration (*saltcon*) was fixed at 0.15 M and the solvent probe radius was set to 1.4 Å. Solvation free energies were computed using the modified generalized Born model 5 (*igb* = 5) as described in Ref. [39]. The nonpolar contribution to solvation was estimated from the assumption of a linear relationship between solvation free energy and solvent-accessible surface area (SASA). Overall, the total binding free energy was computed as

(1) $${∆G}_{bind}={∆E}_{MM}+{∆G}_{solv}-T∆S$$

where ΔE_MM_ represents the gas-phase molecular mechanics energy change, composed of van der Waals (ΔE_vdw_) and electrostatic (ΔE_elect_) terms:

(2) $${∆E}_{MM}\;or\;{∆G}_{gas}={∆E}_{vdw}+{∆E}_{elect}$$

and ΔG_solv_ is the solvation free energy change, given by the sum of polar solvation (ΔG_polar_, from the GB model) and nonpolar solvation (ΔG_nonpolar_, from SASA):

(3) $${∆G}_{solv}={∆G}_{polar}+{∆G}_{nonpolar}$$

This decomposition allowed assessment of the energetic components governing ligand binding in the wild-type EGFR complexes.

## 5. Conclusions

This study demonstrates a successful natural product-guided optimization pipeline that converts a chalcone scaffold from *Syzygium nervosum* into promising lead compounds against non-small cell lung cancer. DMC (**1**) was efficiently isolated and structurally confirmed by HRMS and spectroscopy, providing a well-defined starting point for rational derivatization. Targeted modification at the reactive 4′-hydroxyl group yielded two [(bromomethyl)phenyl]methyl ether derivatives (**2a** and **2b**) in high yields, establishing a practical synthetic route for systematic structure–activity relationship development. Biological evaluation revealed that both derivatives markedly enhanced antiproliferative potency in the NCI-H460 NSCLC model (IC_50_ = 7.91–7.97 μM), outperforming the clinically used EGFR inhibitor osimertinib under the same conditions, while showing weak activity in A549 cells. Importantly, the derivatives, particularly **2a**, exhibited reduced cytotoxicity toward normal lung fibroblasts (MRC-5) compared with osimertinib, highlighting an improved in vitro therapeutic window and positioning these compounds as selective lead candidates. Computational analyses provided a coherent mechanistic basis for the observed activity gains. DFT-derived descriptors indicated that **2a**/**2b** possess increased electronic stability (larger HOMO–LUMO gaps; greater hardness), consistent with binding modes driven by shape complementarity and localized hydrogen bonding. MEP mapping localized electron-rich regions at oxygen functionalities (and around Br), rationalizing the enhanced noncovalent interaction potential within the binding pocket. Docking against wild-type EGFR (PDB ID: 1M17) showed improved predicted affinities for **2a**/**2b** relative to DMC and scores comparable to osimertinib, supported by a validated protocol (redocking RMSD ~1.23 Å). Long-timescale MD simulations further distinguished the leads: **2a** and **2b** maintained more persistent binding poses than osimertinib, and MM/GBSA identified **2a** as the strongest binder with favorable dispersion-driven stabilization and a lower desolvation penalty. Collectively, these results establish **2a** and **2b** as impactful EGFR-oriented chalcone leads and provide an integrated experimental computational framework to guide further optimization, mechanistic validation and in vivo development.

## Figures and Tables

**Table 1:** Antiproliferative activity of [(bromomethyl)phenyl]methyl–DMC derivatives (**2a** and **2b**) against non-small cell lung cancer cell lines (A549 and NCI-H460) and normal human lung fibroblasts (MRC-5).

Compounds	Half-Maximal Inhibitory Concentration (IC_50_, µM) ^a^			Selectivity Index (SI)	
	MRC-5	A549	NCI-H460	A549	NCI-H460
DMC (**1**)	11.45 ± 0.71	14.74 ± 2.57	20.80 ± 2.02	0.78	0.55
Compound **2a**	≥50	≥50	7.97 ± 0.62	ND	≥6.26
Compound **2b**	16.08 ± 0.45	≥50	7.91 ± 2.77	≤0.32	2.88
Osimertinib (+) ^b^	3.43 ± 0.35	2.25 ± 0.21	14.10 ± 0.35	1.52	0.24

^a^ Expressed as the mean ± standard deviation (SD) of triplicate experiments. ^b^ Positive control (+). ND = Not determined. IC_50_, half-maximal inhibitory concentration. The SI value suggests potential anti-lung cancer activity, with a cutoff value greater than 2. SI was not determined when IC_50_ exceeded 50 µM.

**Table 2:** Frontier molecular orbitals (HOMO/LUMO) of DMC (**1**), **2a**, **2b** and osimertinib were computed by DFT under different solvent conditions to assess solvent effects on their electronic properties.

Compounds	Solvent	DFT-B3LYP/6–31G(d,p)		
		Normal	HOMO	LUMO
DMC (**1**)	Water	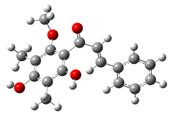	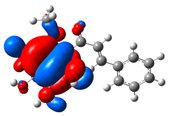	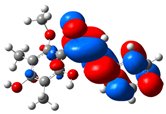
	DMSO	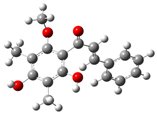	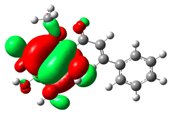	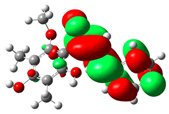
Compound **2a**	Water	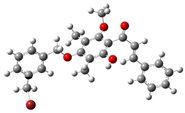	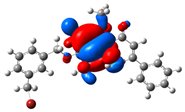	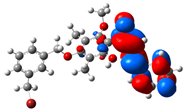
	DMSO	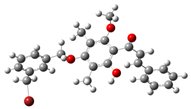	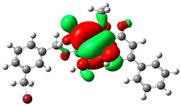	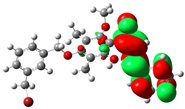
Compound **2b**	Water	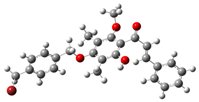	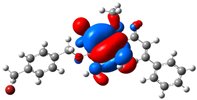	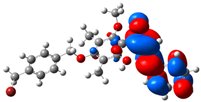
	DMSO	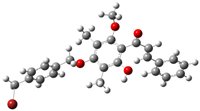	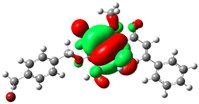	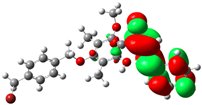
Osimertinib	Water	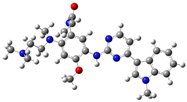	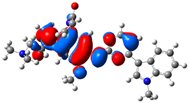	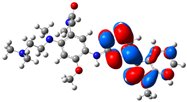
	DMSO	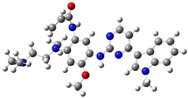	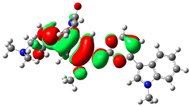	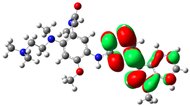

**Table 3:** Calculated energy values of DMC (**1**), DMC derivatives (**2a** and **2b**) and osimertinib by DFT-B3LYP/6–31G(d,p) method.

Parameters	DFT-B3LYP/6–31G(d,p)							
	DMC (1)		DMC Derivative (2a)		DMC Derivative (2b)		Osimertinib	
	Water	DMSO	Water	DMSO	Water	DMSO	Water	DMSO
E_HOMO_ (eV)	−5.8858	−5.8042	−5.8940	−5.8069	−5.8940	−5.8069	−4.9770	−4.9443
E_LUMO_ (eV)	−2.0381	−1.8694	−2.0381	−1.8694	−2.0381	−1.8694	−1.1102	−1.1048
ΔE (eV)	3.8477	3.9348	3.8559	3.9375	3.8559	3.9375	3.8668	3.8395
η (eV)	1.9239	1.9674	1.9280	1.9688	1.9280	1.9688	1.9334	1.9198
s (eV^−1^)	0.2599	0.2541	0.2593	0.2540	0.2593	0.2540	0.2586	0.2604
ω^−^	6.3010	5.9056	6.3034	5.9065	6.3034	5.9065	4.1591	4.1348
ω^+^	2.3391	2.0688	2.3373	2.0683	2.3373	2.0683	1.1155	1.1103

E_HOMO_ = Highest occupied molecular orbital energy. E_LUMO_ = Lowest unoccupied molecular orbital energy. ΔE = HOMO–LUMO energy gap. η = Chemical hardness. s = Chemical softness. ω^−^ = Electrodonating power. ω^+^ = Electroaccepting power. The equations used for the calculations are provided in the Supporting Information.

**Table 4:** Molecular electrostatic potential (MEP) surfaces of DMC (**1**), **2a**, **2b** and osimertinib under different solvent environments, calculated using DFT methods.

Compounds	DFT-B3LYP/6–31G(d,p)	
	Water	DMSO
DMC (**1**)	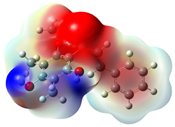	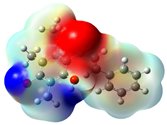
Compound **2a**	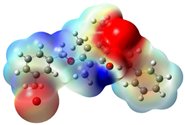	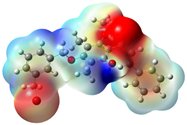
Compound **2b**	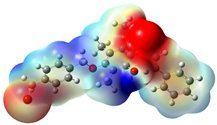	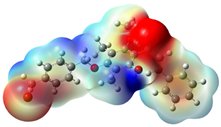
Osimertinib	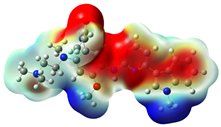	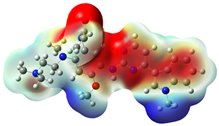

**Table 5:** Docking results of the test ligands at the binding site of the wild-type EGFR (PDB ID: 1M17).

Compounds	Amino Acid Residues Interactions			ΔG (kcal mol^−1^)	Binding Constant (Ka) (M^−1^)
	Conventional Hydrogen Bond	π–Sulfur, π–Anion, π–Sigma and π–π Stacked	Hydrophobic		
DMC (**1**)	-	Met742	Leu694, Gly695, Phe699, Val702, Ala719, Ile720, Lys721, Gly738, Leu753, Leu764, Ile765, Thr766, Leu768, Met769, Gly772, Cys773, Leu820, Thr830, Asp831	−7.7	4.43 × 10^5^
Compound **2a**	Leu694, Gly772	Val702, Glu780	Gly695, Ala719, Lys721, Met742, Thr766, Leu768, Met769, Pro770, Phe771, Cys773, Asp776, Tyr777, His781, Leu820, Asp831	−8.6	2.02 × 10^6^
Compound **2b**	Gly772	Leu694, Glu780, Leu820	Gly695, Val702, Ala719, Lys721, Met742, Cys751, Leu764, Thr766, Leu768, Met769, Pro770, Phe771, Cys773, Asp776, Tyr777, His781, Thr830, Asp831	−8.8	2.84 × 10^6^
Osimertinib	Lys721	Phe699, Val702, Glu738, Asp831	Leu694, Ala719, Ile720, Leu723, Ala731, Glu734, Ile735, Met742, Thr766, Gln767, Leu768, Met769, Pro770, Gly772, Cys773, Leu820, Thr830, Gly833, Leu834, Lys851	−8.5	1.72 × 10^6^

**Table 6:** Summary of the hydrogen-bond lengths and intermolecular distances among DMC derivatives involved in stabilizing the docked complexes.

Compounds	Hydrogen-Bond Lengths (Å)		
	Leu694	Gly722	Lys721
DMC (**1**)	-	-	-
Compound **2a**	2.14	2.81	-
Compound **2b**	-	2.84	-
Osimertinib	-	-	2.23

**Table 7:** MM/GBSA-calculated binding free energies and associated energy components.

Components	Free Energy (kcal/mol)		
	Compound 2a	Compound 2b	Osimertinib
Gas phase			
ΔE_vdw_	−49.61 ± 3.89	−40.39 ± 5.93	−41.76 ± 5.44
ΔE_elect_	−5.62 ± 4.17	−11.32 ± 6.44	−13.86 ± 5.48
Solution phase			
ΔG_polar_	18.75 ± 3.51	26.80 ± 6.20	28.25 ± 4.72
ΔG_nonpolar_	−5.47 ± 0.29	−4.91 ± 0.65	−4.56 ± 0.54
Binding energy			
ΔG_gas_	−55.23 ± 4.69	−51.71 ± 7.10	−55.62 ± 8.77
ΔG_solv_	13.27 ± 3.48	21.89 ± 6.30	23.69 ± 4.44
ΔG_total_	−41.96 ± 3.38	−29.82 ± 5.75	−31.93 ± 6.11
−TΔS	13.18 ± 5.09	7.83 ± 7.42	19.74 ± 1.76
ΔG_bind_	−28.78 ± 6.11	−21.99 ± 9.39	−12.19 ± 6.36

## Data Availability

The data supporting the findings of this study are available from the corresponding author upon reasonable request.

## Supplementary Materials

The following supporting information can be downloaded at: https://www.mdpi.com/article/10.3390/ijms27146104/s1.
