# Supplementary material for: [(Bromomethyl)phenyl]methyl-Conjugated Chalcone Derivatives as Potential Lung Cancer Inhibitors: Structure Modification, Molecular Docking, Molecular Dynamics and In Vitro Validation
Source: Int J Mol Sci. 2026 Jul 8;27(14):6104. doi: 10.3390/ijms27146104 (PMC13410135; doi:10.3390/ijms27146104)
Supplement: Supplementary file 1 [file ijms-27-06104-s001.zip › ijms-4396995-supplementary.pdf]

[(Bromomethyl)phenyl]methyl-Conjugated Chalcone Derivatives as Potential Lung Cancer Inhibitors: Structure Modification, Molecular Docking, Molecular Dynamics and *In Vitro* Validation

Nathaporn Cheechana [1,2], Nopawit Khamto [3], Kraikrit Utama [1,2], Chawanakorn Kongsak [1,2], Puracheth Rithchumpon [4], Padchanee Sangthong [1,5,6] and Puttinan Meepowpan [1,5*]

[1]*Department of Chemistry, Faculty of Science, Chiang Mai University, 239 Huay Kaew Road, Chiang Mai 50200, Thailand.*

[2]*Office of Research Administration, Chiang Mai University, Chiang Mai 50200, Thailand.*

[3]*Department of Biochemistry, Faculty of Medical Science, Naresuan University, Phitsanulok, 65000, Thailand.*

[4]*Department of Chemistry, Faculty of Science, Khon Kaen University, Khon Kaen 40002, Thailand.*

[5]*Center of Excellence in Materials Science and Technology, Chiang Mai University, 239 Huay Kaew Road, Chiang Mai 50200, Thailand.*

[6]*Division of Biochemistry and Biochemical Innovation, Department of Chemistry, Faculty of Science, Chiang Mai University, Chiang Mai, 50200, Thailand.*

**Corresponding email: puttinan.m@cmu.ac.th*

Table of contents

1. ^{1}H-NMR, ^{13}C-NMR, FTIR and ESI-MS spectra

^{1}H-NMR spectrum of 2′,4′-Dihydroxy-6′-methoxy-3′,5′-dimethylchalcone (DMC) (**1**) 3

^{1}H-NMR spectrum of 2′-hydroxy-4′-(3-(bromomethyl)benzyloxy)-6′-methoxy-3′,5′-dimethylchalcone (**2a**) 3

^{1}H-NMR spectrum of 2′-hydroxy-4′-(4-(bromomethyl)benzyloxy)-6′-methoxy-3′,5′-dimethylchalcone (**2b**) 4

^{13}C-NMR spectrum of 2′,4′-Dihydroxy-6′-methoxy-3′,5′-dimethylchalcone (DMC) (**1**) 4

^{13}C-NMR spectrum of 2′-hydroxy-4′-(3-(bromomethyl)benzyloxy)-6′-methoxy-3′,5′-dimethylchalcone (**2a**) 5

^{13}C-NMR spectrum of 2′-hydroxy-4′-(4-(bromomethyl)benzyloxy)-6′-methoxy-3′,5′-dimethylchalcone (**2b**) 5

FTIR spectrum of 2′,4′-Dihydroxy-6′-methoxy-3′,5′-dimethylchalcone (DMC) (**1**) 6

FTIR spectrum of 2′-hydroxy-4′-(3-(bromomethyl)benzyloxy)-6′-methoxy-3′,5′-dimethylchalcone (**2a**) 6

FTIR spectrum of 2′-hydroxy-4′-(4-(bromomethyl)benzyloxy)-6′-methoxy-3′,5′-dimethylchalcone (**2b**) 7

ESI-MS spectrum of 2′,4′-Dihydroxy-6′-methoxy-3′,5′-dimethylchalcone (DMC) (**1**) 7

ESI-MS spectrum of 2′-hydroxy-4′-(3-(bromomethyl)benzyloxy)-6′-methoxy-3′,5′-dimethylchalcone (**2a**) 8

ESI-MS spectrum of 2′-hydroxy-4′-(4-(bromomethyl)benzyloxy)-6′-methoxy-3′,5′-dimethylchalcone (**2b**) 8

Table of contents (continue)

2. The equations used to calculate the results presented in Table 3 9

3. Density Functional Theory Calculations

2′,4′-Dihydroxy-6′-methoxy-3′,5′-dimethylchalcone (DMC) (**1**) 10

2′-hydroxy-4′-(3-(bromomethyl)benzyloxy)-6′-methoxy-3′,5′-dimethylchalcone (**2a**) 11

2′-hydroxy-4′-(4-(bromomethyl)benzyloxy)-6′-methoxy-3′,5′-dimethylchalcone (**2b**) 13

Osimertinib 15

1. ^{1}H-NMR, ^{13}C-NMR, FTIR and HRMS-ESI spectra

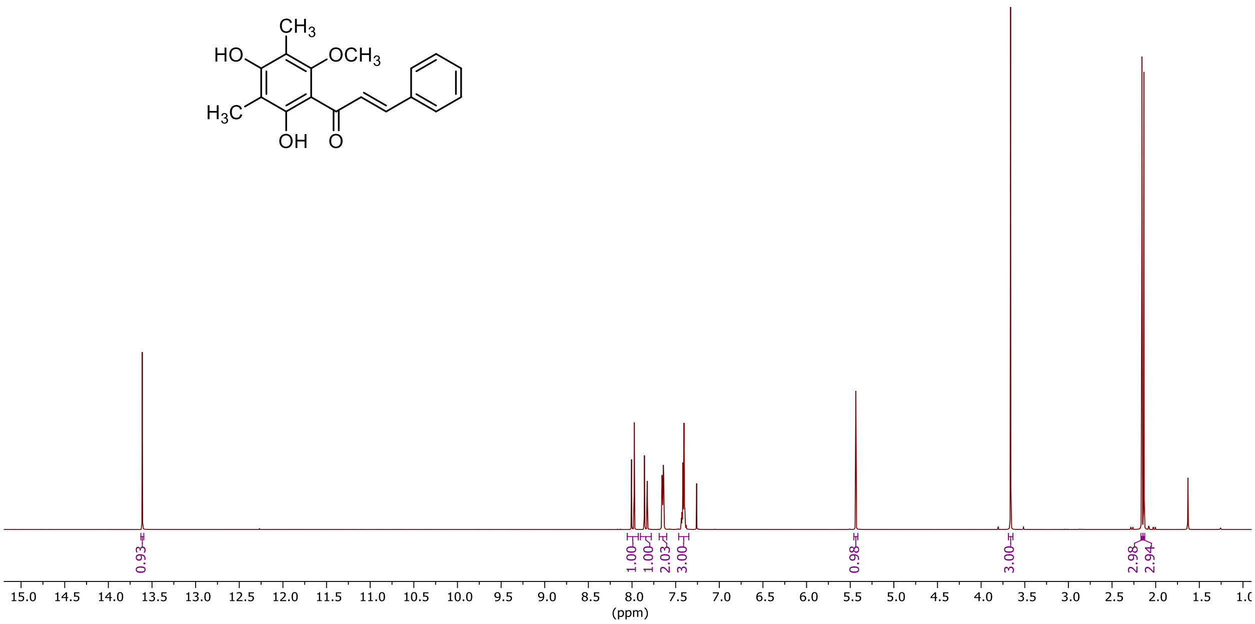

Figure S1 ^{1}H-NMR (500 MHz, $CDCl_3$) spectrum of 2′,4′-Dihydroxy-6′-methoxy-3′,5′-dimethylchalcone (DMC) (**1**)

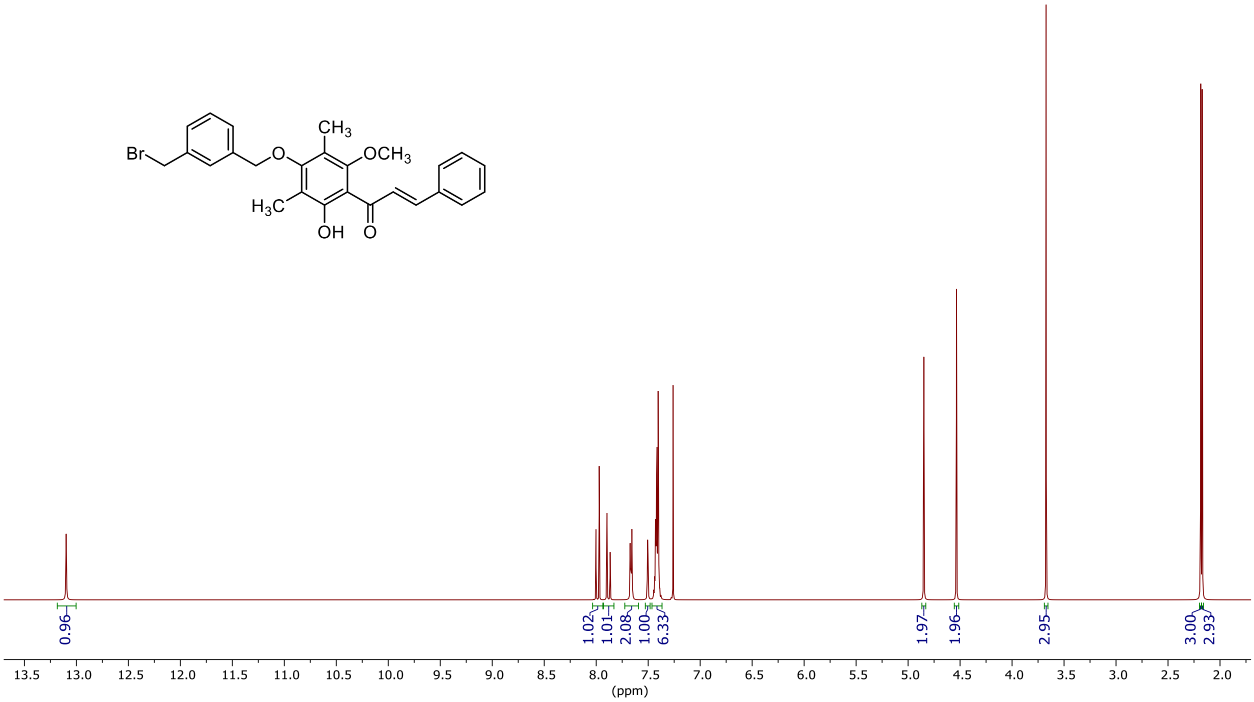

Figure S2 ^{1}H-NMR (500 MHz, $CDCl_3$) spectrum of 2′-hydroxy-4′-(3-(bromomethyl)benzyloxy)-6′-methoxy-3′,5′-dimethylchalcone (**2a**)

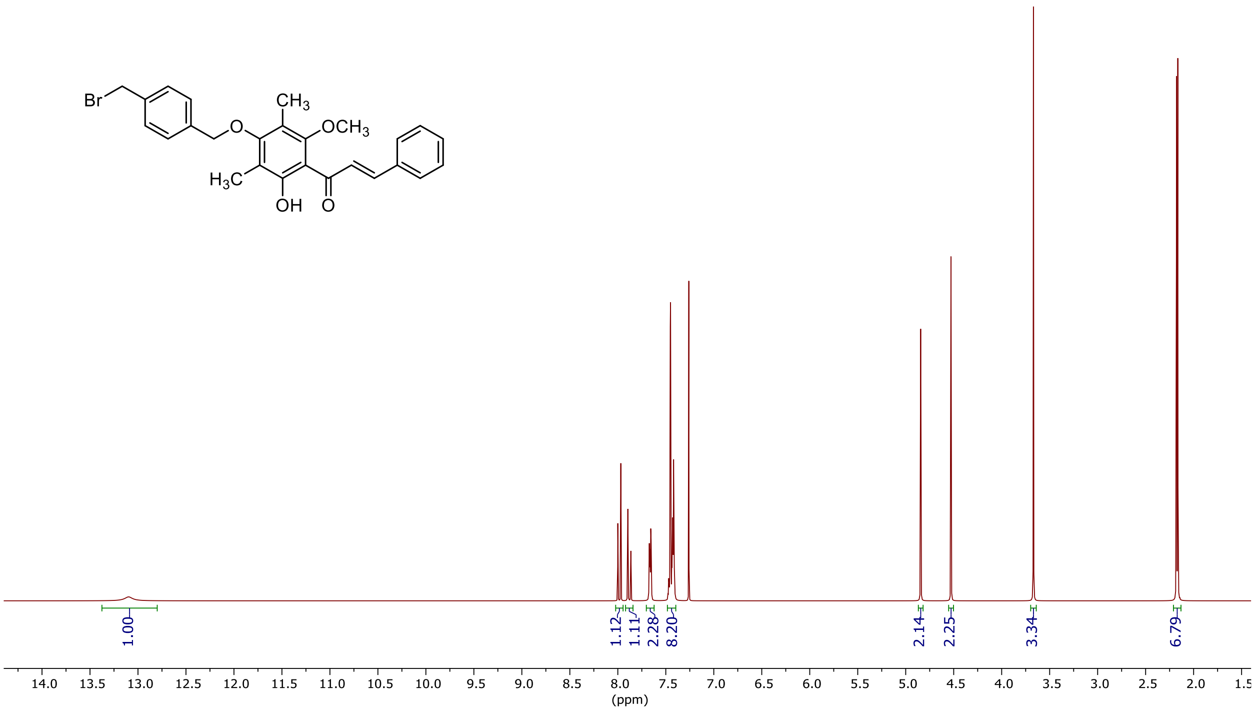

Figure S3 ^{1}H-NMR (500 MHz, $CDCl_3$) spectrum of 2′-hydroxy-4′-(4-(bromomethyl)benzyloxy)-6′-methoxy-3′,5′-dimethylchalcone (**2b**)

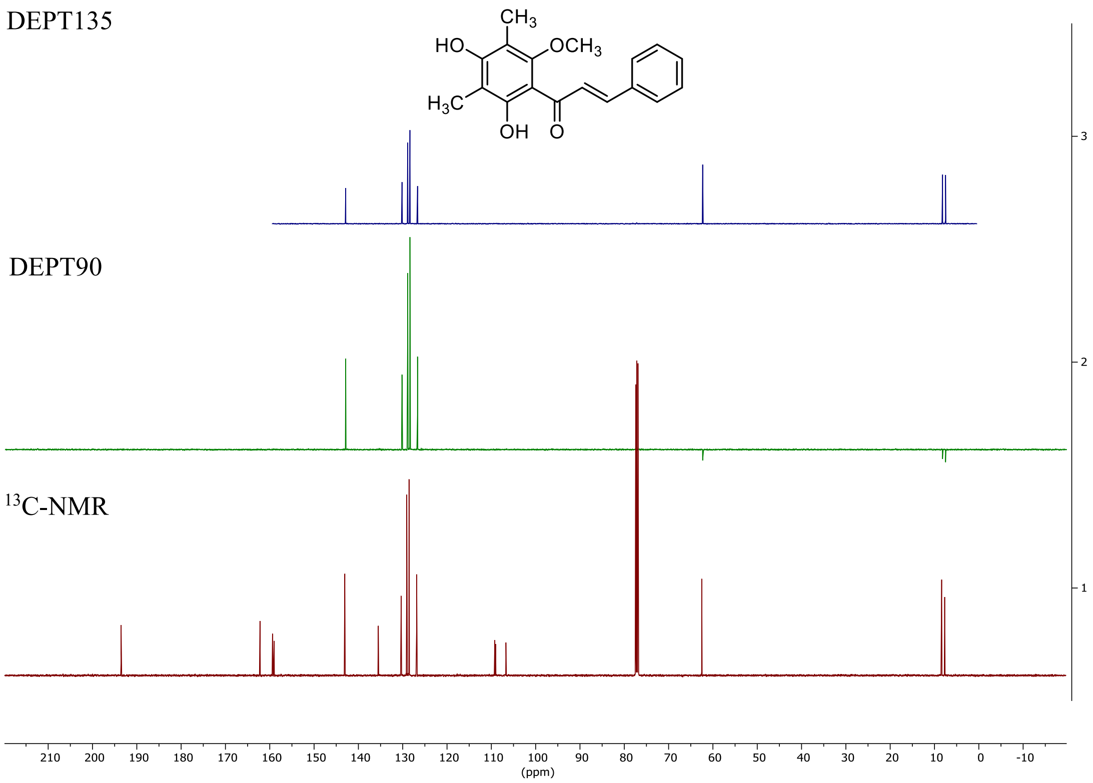

Figure S4 ^{13}C-NMR (125 MHz, $CDCl_3$) spectrum of 2′,4′-Dihydroxy-6′-methoxy-3′,5′-dimethylchalcone (DMC) (**1**)

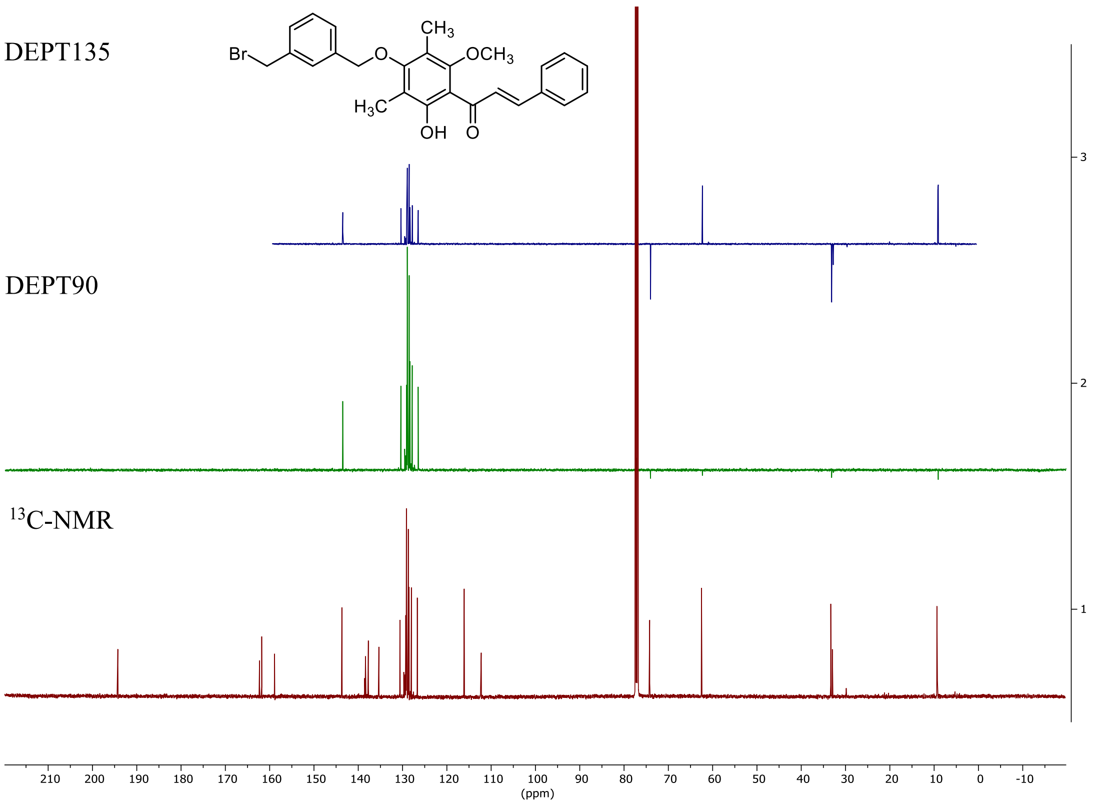

Figure S5 ^{13}C-NMR (125 MHz, $CDCl_3$) spectrum of 2′-hydroxy-4′-(3-(bromomethyl)benzyloxy)-6′-methoxy-3′,5′-dimethylchalcone (**2a**)

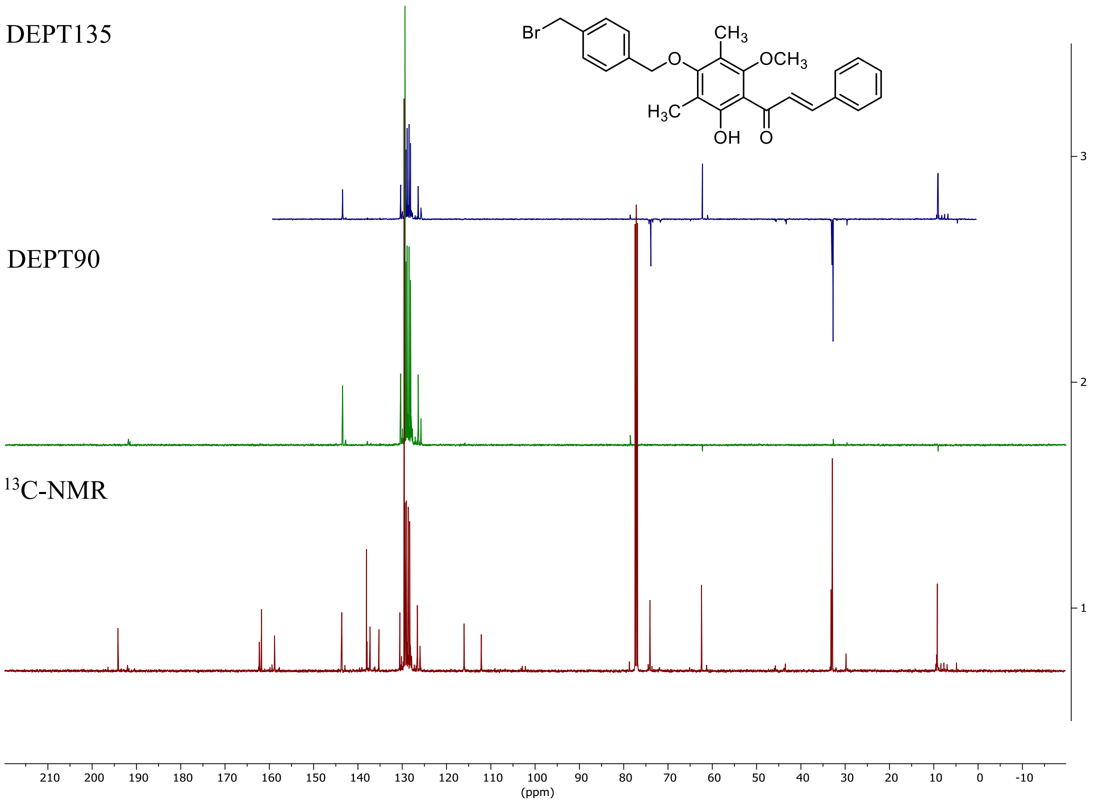

Figure S6 ^{13}C-NMR (125 MHz, $CDCl_3$) spectrum of 2′-hydroxy-4′-(4-(bromomethyl)benzyloxy)-6′-methoxy-3′,5′-dimethylchalcone (**2b**)

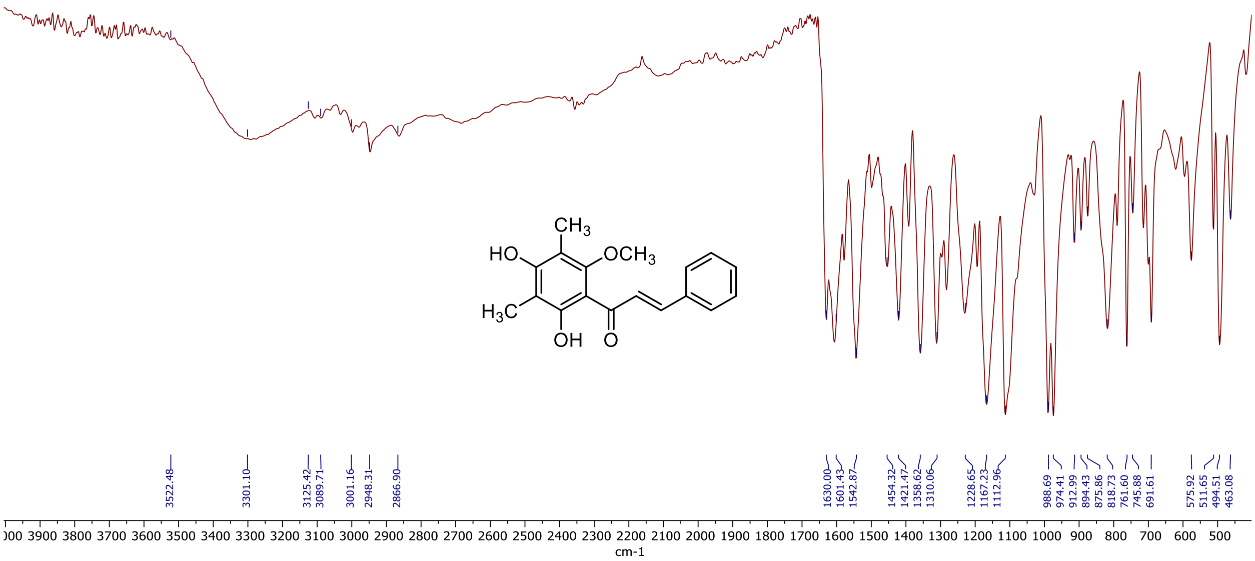

Figure S7 FTIR (ATR) spectrum of 2′,4′-Dihydroxy-6′-methoxy-3′,5′-dimethylchalcone (DMC) (**1**)

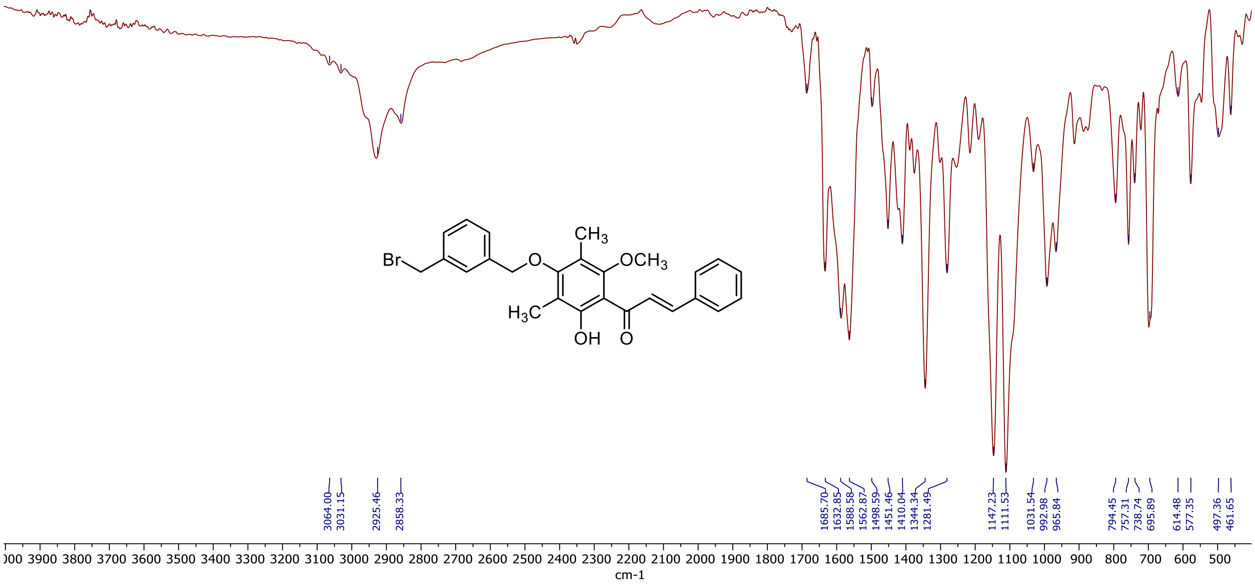

Figure S8 FTIR (ATR) spectrum of 2′-hydroxy-4′-(3-(bromomethyl)benzyloxy)-6′-methoxy-3′,5′-dimethylchalcone (**2a**)

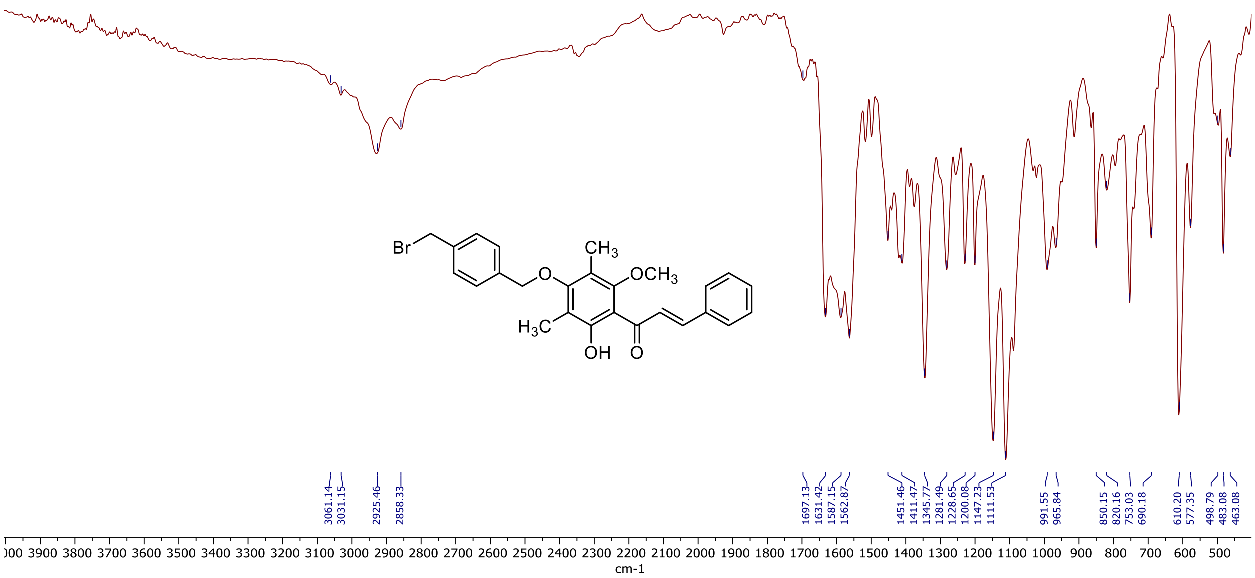

Figure S9 FTIR (ATR) spectrum of 2′-hydroxy-4′-(4-(bromomethyl)benzyloxy)-6′-methoxy-3′,5′-dimethylchalcone (**2b**)

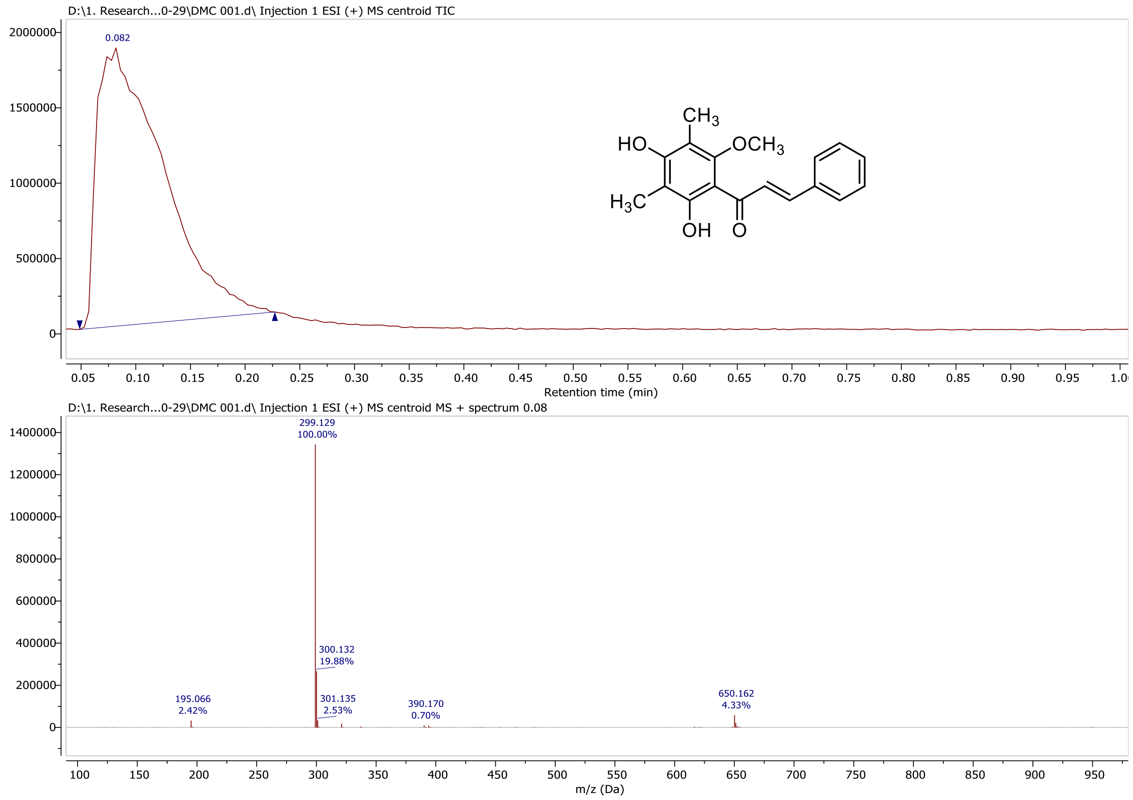

Figure S10 HRMS-ESI spectrum of 2′,4′-Dihydroxy-6′-methoxy-3′,5′-dimethylchalcone (DMC) (**1**)

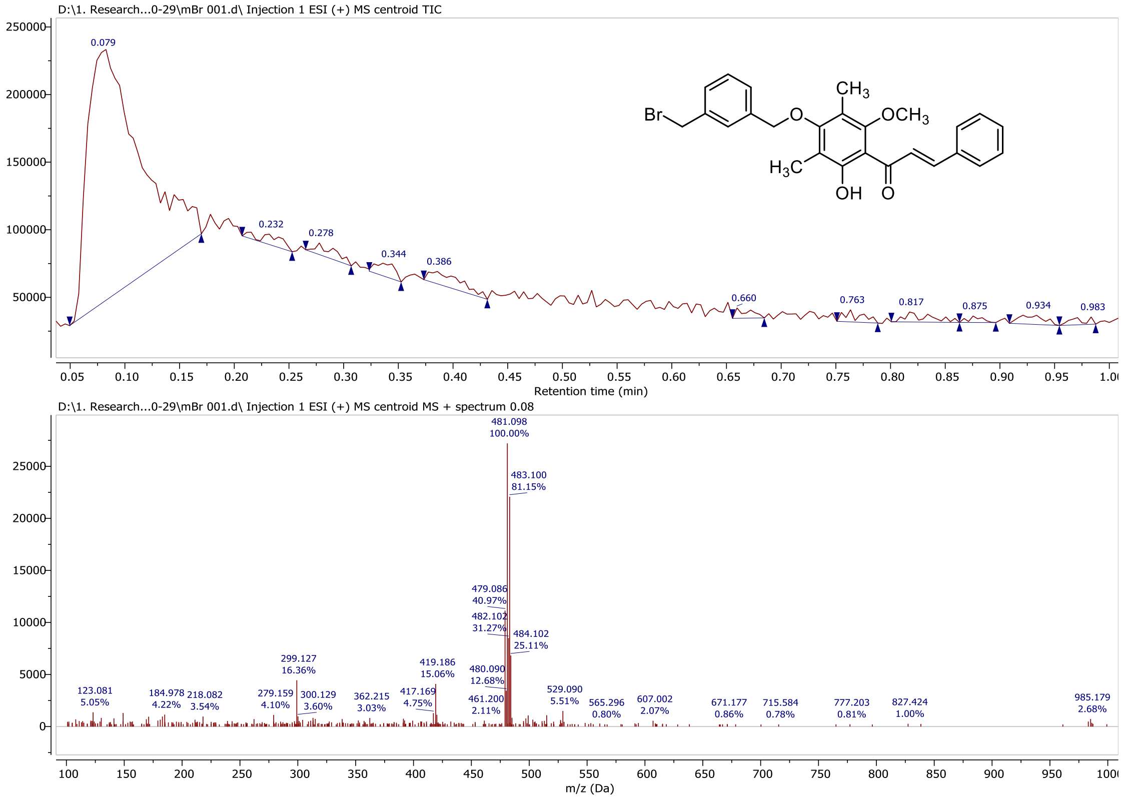

Figure S11 HRMS-ESI spectrum of 2′-hydroxy-4′-(3-(bromomethyl)benzyloxy)-6′-methoxy-3′,5′-dimethylchalcone (**2a**)

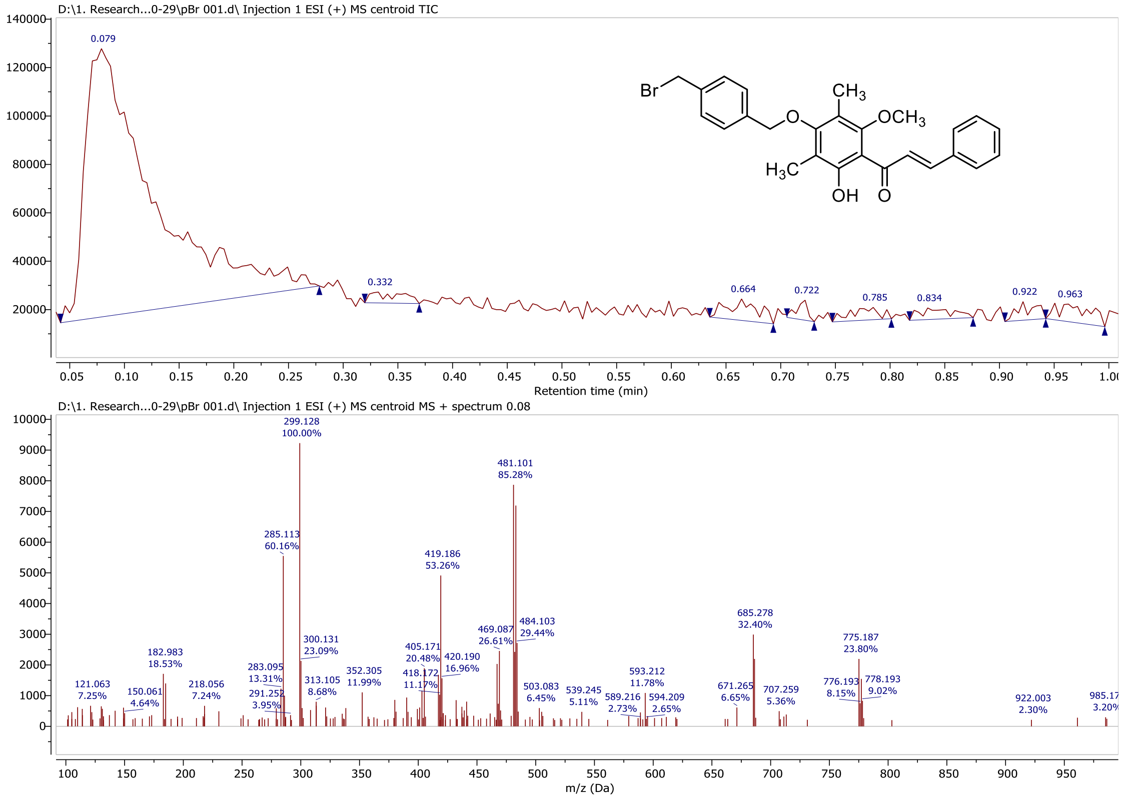

Figure S12 HRMS-ESI spectrum of 2′-hydroxy-4′-(4-(bromomethyl)benzyloxy)-6′-methoxy-3′,5′-dimethylchalcone (**2b**)

2. The equations used to calculate the results presented in Table 3

From the Gaussian output file, the HOMO–LUMO energy values are obtained in Hartree. Therefore, the energies must be converted from Hartree to eV before calculating the related parameters. The unit conversion is performed using **Equation (1)**.

$$1 \text{ Hartree} = 27.2114 \text{ eV} \tag{1}$$

The HOMO–LUMO energy gap (ΔE) can be calculated using **Equation (2)**.

$$\Delta E(\text{eV}) = E_{\text{LUMO}}(\text{eV}) - E_{\text{HOMO}}(\text{eV}) \tag{2}$$

Chemical hardness (η) can be calculated using **Equation (3)**.

$$\text{Chemical hardness } (\eta) = \left[\frac{I - A}{2}\right] \tag{3}$$

$I \approx -E_{HOMO}$, $A \approx -E_{LUMO}$

Chemical softness (s) can be calculated using **Equation (4)**.

$$\text{Chemical softness } (s) = \left[\frac{1}{2\eta}\right] \tag{4}$$

Electrodonating power (ω^-) can be calculated using **Equation (5)**.

$$\text{Electrodonating power } (\omega^-) = \frac{(3I + A)^2}{16(I - A)} \tag{5}$$

Electroaccepting power (ω^+) can be calculated using **Equation (6)**.

$$\text{Electroaccepting power } (\omega^+) = \frac{(I + 3A)^2}{16(I - A)} \tag{6}$$

3. Density Functional Theory Calculations

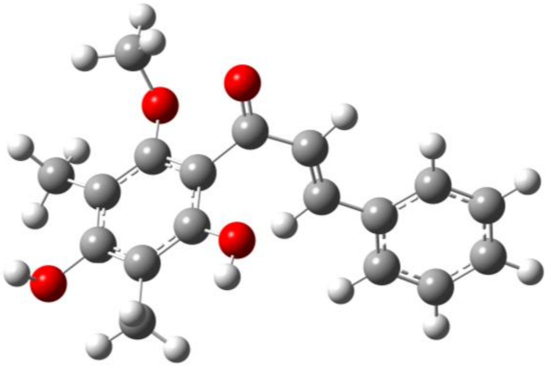

2′,4′-Dihydroxy-6′-methoxy-3′,5′-dimethylchalcone (DMC) (**1**)

Symbol	Bond	Angle	Dihedral	X	Y	Z
C				-1.94307	0.90386	-0.40532
C	1.401327			-1.08127	0.449474	0.601927
C	1.400879	118.6123		-1.24869	-0.85248	1.091167
C	1.404365	121.6542	0.122309	-2.25055	-1.70281	0.595733
C	1.402816	117.5285	0.095757	-3.08423	-1.20228	-0.41538
C	1.403406	122.9872	0.504756	-2.94939	0.089431	-0.94721
C	1.514645	119.8806	-177.47	-0.04935	1.38801	1.192227
C	1.472261	119.791	-111.977	1.382273	1.150556	0.944
H	1.086676	113.3674	-175.494	2.032218	1.838315	1.478251
C	1.347748	124.3784	6.900758	1.863721	0.21345	0.103486
H	1.088374	117.5315	-1.05212	1.142712	-0.41855	-0.41157
C	1.463755	127.46	178.3858	3.265635	-0.05837	-0.21796
C	1.408629	123.2077	2.562056	4.341131	0.627755	0.379344
C	1.406894	118.646	-177.531	3.559028	-1.05386	-1.16785
C	1.390361	120.7218	-179.983	5.654263	0.327611	0.034812
H	1.08513	119.8483	0.196479	4.146606	1.397577	1.118967
C	1.39304	121.1401	179.9499	4.874604	-1.35431	-1.5136
H	1.08687	119.0376	0.076841	2.740209	-1.59248	-1.63764
C	1.395122	119.9515	0.076233	5.92768	-0.66385	-0.91305
H	1.086033	119.6621	-179.964	6.47038	0.866962	0.506533
H	1.085954	119.8598	-179.921	5.077252	-2.12589	-2.25042
H	1.085934	120.2157	179.9706	6.95487	-0.89492	-1.17902
O	1.227077	120.0503	67.03972	-0.40731	2.353232	1.860007
O	1.379036	118.4464	-176.462	-1.729	2.158389	-0.93643

Symbol	Bond	Angle	Dihedral	X	Y	Z
C	1.508084	121.6529	175.9733	-3.80374	0.565874	-2.09499
H	1.097733	112.3727	92.84272	-4.70892	1.0853	-1.7546
H	1.092089	110.0272	-147.881	-3.24183	1.26996	-2.71239
H	1.092197	110.4777	-27.1418	-4.1225	-0.27839	-2.71022
C	1.509726	120.5081	179.1396	-2.39554	-3.10804	1.128254
H	1.098622	112.2726	66.76626	-2.71378	-3.12413	2.179651
H	1.089919	110.4291	-173.005	-3.13588	-3.65983	0.549166
H	1.098398	111.6341	-54.1033	-1.45234	-3.66667	1.059011
C	1.434838	114.9452	-95.8856	-2.5182	3.205173	-0.35319
H	1.097253	111.2692	-61.0122	-3.59067	3.019821	-0.49252
H	1.09349	110.3309	61.43626	-2.29019	3.304549	0.711638
H	1.09178	106.0689	-179.565	-2.24212	4.120897	-0.8797
O	1.368657	116.8436	-179.437	-0.39509	-1.25471	2.082522
H	0.965444	109.4447	177.3832	-0.63009	-2.147	2.366544
O	1.43	118.0761	177.2787	-4.06972	-2.07762	-0.9699
H	0.96	109.5	91.7137	-4.89299	-1.98103	-0.48566

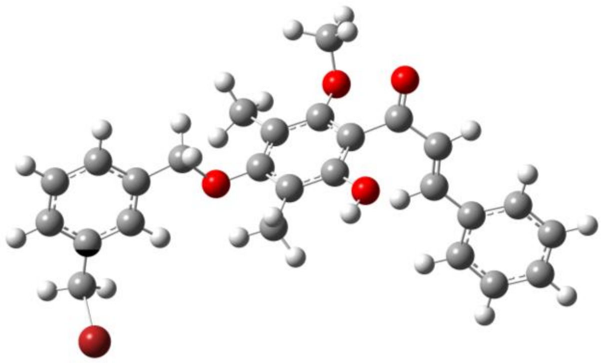

2′-hydroxy-4′-(3-(bromomethyl)benzyloxy)-6′-methoxy-3′,5′-dimethylchalcone (**2a**)

Symbol	Bond	Angle	Dihedral	X	Y	Z
C				-1.88343	-2.06405	-0.59256
C	1.401327			-2.54227	-1.46097	0.487224
C	1.400879	118.6123		-1.79675	-0.66993	1.370924
C	1.404366	121.6542	0.122263	-0.41775	-0.46953	1.196485
C	1.402816	117.5285	0.095811	0.193872	-1.0911	0.097641
C	1.403407	122.9872	0.504698	-0.51089	-1.88256	-0.82239
C	1.514645	119.8806	-177.47	-4.01305	-1.73583	0.722618

Symbol	Bond	Angle	Dihedral	X	Y	Z
C	1.472261	119.791	-111.977	-4.99831	-0.65878	0.530877
H	1.086676	113.3674	-175.494	-6.01033	-0.94711	0.802101
C	1.347747	124.3784	6.900824	-4.70896	0.55586	0.02358
H	1.088374	117.5315	-1.05214	-3.67338	0.754919	-0.24569
C	1.463755	127.46	178.3858	-5.63644	1.660462	-0.22585
C	1.408629	123.2077	2.562098	-7.0067	1.604897	0.09594
C	1.406894	118.646	-177.531	-5.14002	2.838278	-0.81378
C	1.390361	120.7218	-179.983	-7.84181	2.685627	-0.16427
H	1.085131	119.8483	0.196415	-7.41795	0.711612	0.554658
C	1.393039	121.1401	179.9499	-5.97671	3.920972	-1.0751
H	1.08687	119.0376	0.076837	-4.08488	2.896912	-1.06781
C	1.395122	119.9515	0.076232	-7.33174	3.848223	-0.75112
H	1.086033	119.6621	-179.964	-8.89554	2.624383	0.091422
H	1.085953	119.8599	-179.921	-5.57168	4.819638	-1.53079
H	1.085934	120.2157	179.9706	-7.98775	4.689855	-0.9525
O	1.227076	120.0503	67.03976	-4.38307	-2.85773	1.0545
O	1.379036	118.4464	-176.462	-2.63257	-2.78085	-1.50181
C	1.508084	121.6529	175.9733	0.158477	-2.46358	-2.04251
H	1.097733	112.3726	92.84282	0.54307	-3.47641	-1.86565
H	1.092089	110.0271	-147.881	-0.55651	-2.53016	-2.86532
H	1.092195	110.4777	-27.1418	1.002648	-1.84148	-2.3479
C	1.509727	120.508	179.1396	0.356927	0.403899	2.153709
H	1.098621	112.2726	66.76626	0.410783	-0.02911	3.16196
H	1.089918	110.4291	-173.005	1.377602	0.545409	1.798576
H	1.098398	111.6341	-54.1033	-0.09243	1.401978	2.245356
O	1.387604	118.0762	177.2786	1.543938	-0.85438	-0.11853
C	1.42748	114.8542	97.3815	2.418599	-1.86139	0.389993
H	1.099379	110.1178	61.51539	2.212997	-2.82362	-0.10038
H	1.100398	109.6927	-55.9304	2.234672	-2.00364	1.465545
C	1.51205	109.9639	-177.04	3.857917	-1.4592	0.159982
C	1.401712	119.068	-177.98	4.880416	-2.33519	0.54977
C	1.394184	121.7911	2.28998	4.195237	-0.23895	-0.42392
C	1.39275	120.4369	-179.603	6.216138	-1.99	0.358881

Symbol	Bond	Angle	Dihedral	X	Y	Z
H	1.087614	119.8055	0.475361	4.629467	-3.29142	1.003151
C	1.402544	120.8134	179.7959	5.537532	0.115185	-0.62383
H	1.08437	119.1614	-0.21922	3.404519	0.439339	-0.72483
C	1.395045	120.1409	-0.07286	6.547031	-0.76689	-0.22478
H	1.085862	119.9016	-179.941	7.001246	-2.67562	0.66322
H	1.086533	120.1379	179.7837	7.589151	-0.49612	-0.37047
C	1.492823	120.0511	179.3701	5.879128	1.420058	-1.26347
H	1.088227	112.9703	-156.628	6.863934	1.421133	-1.72649
H	1.088242	112.9667	-30.4998	5.12639	1.754213	-1.9748
Br	2.008298	111.8764	86.27979	5.967898	2.911012	0.079082
C	1.434837	114.9453	-95.8856	-2.67885	-4.19547	-1.26628
H	1.097252	111.2692	-61.0122	-1.67685	-4.64	-1.31454
H	1.09349	110.3308	61.43627	-3.13511	-4.40412	-0.29468
H	1.09178	106.0689	-179.565	-3.29494	-4.61468	-2.0642
O	1.368657	116.8436	-179.437	-2.47409	-0.10811	2.419157
H	0.965444	109.4447	177.3832	-1.85486	0.376908	2.978969

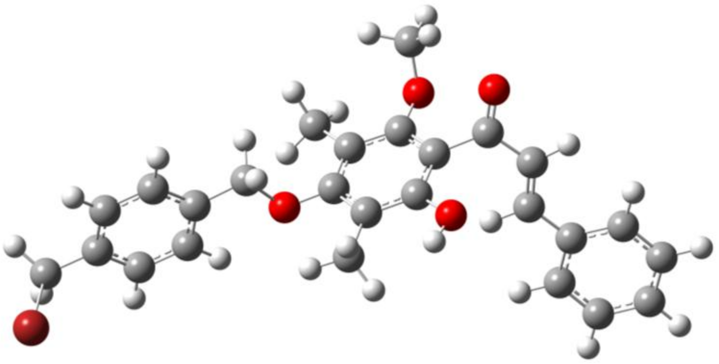

2′-hydroxy-4′-(4-(bromomethyl)benzyloxy)-6′-methoxy-3′,5′-dimethylchalcone (**2b**)

Symbol	Bond	Angle	Dihedral	X	Y	Z
C				2.232436	1.906477	-0.44972
C	1.401392			2.893494	1.244568	0.59373
C	1.4009	118.6066		2.178253	0.311967	1.356098
C	1.404278	121.6406	0.096191	0.82743	0.029243	1.096599
C	1.402848	117.5579	0.091765	0.212334	0.71534	0.038814
C	1.403455	122.9694	0.551538	0.888667	1.648512	-0.76209
C	1.514633	119.8712	-177.385	4.326821	1.603603	0.926569

Symbol	Bond	Angle	Dihedral	X	Y	Z
C	1.47226	119.8137	-112.147	5.402165	0.629306	0.677711
H	1.086674	113.3571	-175.482	6.375751	0.964012	1.025507
C	1.347728	124.3922	6.907291	5.232362	-0.54538	0.039243
H	1.088379	117.5357	-1.04322	4.228118	-0.79363	-0.29904
C	1.463763	127.4515	178.4012	6.255081	-1.54528	-0.27198
C	1.408631	123.2043	2.594794	7.601764	-1.41963	0.121591
C	1.406895	118.6517	-177.504	5.879891	-2.6907	-0.99765
C	1.390359	120.7229	-179.991	8.531072	-2.40173	-0.20239
H	1.085124	119.8492	0.190598	7.920445	-0.54963	0.686428
C	1.39304	121.1416	179.9571	6.810964	-3.67452	-1.32286
H	1.086878	119.0349	0.087684	4.844447	-2.80249	-1.30856
C	1.395125	119.9517	0.076799	8.141002	-3.53321	-0.92612
H	1.086029	119.6612	-179.964	9.564826	-2.28755	0.110302
H	1.085959	119.8614	-179.918	6.498852	-4.5497	-1.88495
H	1.085931	120.2169	179.9703	8.870298	-4.29772	-1.17689
O	1.227072	120.0389	66.88018	4.592132	2.710227	1.385588
O	1.378918	118.4388	-176.429	2.958301	2.767764	-1.24515
C	1.508097	121.6916	175.8982	0.225765	2.302286	-1.94847
H	1.0977	112.3855	92.90926	-0.24325	3.26044	-1.68981
H	1.09209	110.0177	-147.817	0.966054	2.50592	-2.72511
H	1.092184	110.5051	-27.0993	-0.55434	1.655251	-2.35547
C	1.509706	120.5593	179.1337	0.085372	-0.99473	1.921247
H	1.098636	112.3088	67.04677	-0.04762	-0.67383	2.963524
H	1.089992	110.3142	-172.756	-0.90389	-1.17463	1.500442
H	1.098404	111.6315	-53.8414	0.60977	-1.95976	1.936125
O	1.38759	117.9627	177.3486	-1.10572	0.40185	-0.26096
C	1.42795	114.8167	97.88775	-2.07481	1.289649	0.297354
H	1.099852	109.9567	60.75757	-1.91384	2.308758	-0.08367
H	1.100067	109.8498	-56.6441	-1.95525	1.333811	1.390012
C	1.511054	109.9315	-177.911	-3.46717	0.81887	-0.05336
C	1.402043	119.1339	172.7235	-4.57091	1.461415	0.525107
C	1.396938	121.9433	-7.99278	-3.68906	-0.22958	-0.94944
C	1.389743	120.6863	179.174	-5.86597	1.066062	0.212219

Symbol	Bond	Angle	Dihedral	X	Y	Z
H	1.087608	119.793	-0.8713	-4.41449	2.277511	1.226829
H	1.083521	119.271	0.878771	-2.84015	-0.73192	-1.39778
C	1.402847	120.5889	0.006864	-6.09354	0.015453	-0.68912
H	1.086659	119.8589	179.4432	-6.71149	1.567462	0.675369
C	1.434907	114.9673	-95.9778	2.882453	4.150036	-0.86762
H	1.09723	111.2728	-60.9734	1.851023	4.520735	-0.91908
H	1.093464	110.3238	61.47755	3.281594	4.290817	0.140608
H	1.09177	106.0643	-179.526	3.494592	4.69437	-1.58939
O	1.368715	116.8561	-179.452	2.855445	-0.30479	2.373154
H	0.965423	109.4433	176.9874	2.253039	-0.88575	2.854433
C	1.395037	120.3032	-179.322	-4.9898	-0.62717	-1.25948
H	1.086914	119.5438	-179.667	-5.14812	-1.448	-1.95415
Br	2.91747	109.0449	138.4912	-8.25542	-1.65935	0.327198
C	1.49165	120.6422	-179.403	-7.48321	-0.40114	-1.03593
H	1.08818	112.8838	159.6667	-7.53802	-0.95499	-1.97102
H	1.088461	113.0743	33.64008	-8.18698	0.4291	-1.04801

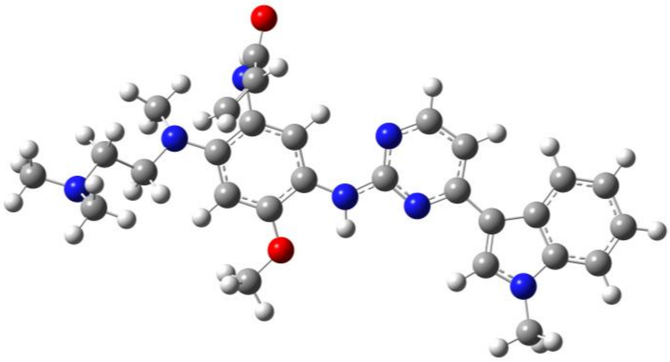

Osimertinib

Symbol	Bond	Angle	Dihedral	X	Y	Z
H				0.940142	-1.45549	-0.34623
C	2.001264			1.751389	0.368552	-0.20547
C	2.29986	117.2258		4.036	0.488701	0.030055
C	2.261659	179.8171	174.2665	2.661733	2.432887	-0.04769
C	1.386189	91.16758	-174.691	3.933007	1.893897	0.074262
H	1.088854	148.385	6.348191	2.515043	3.511512	-0.02212
H	1.080669	120.9937	-177.068	4.793441	2.528117	0.233207

Symbol	Bond	Angle	Dihedral	X	Y	Z
C	2.529957	51.93521	-179.88	-0.6911	-0.23626	-0.46836
C	1.395571	102.1721	179.9081	-1.30439	1.017208	-0.4859
C	1.414693	140.2645	-0.32718	-1.5228	-1.37377	-0.59374
C	1.400123	121.8006	-179.139	-2.69271	1.157796	-0.60065
H	1.080927	118.5992	-0.15542	-0.68469	1.897811	-0.39153
C	1.38985	120.8356	-179.53	-2.89896	-1.2382	-0.73335
C	1.408832	120.676	-1.46735	-3.52115	0.026126	-0.7341
H	1.080995	119.1854	177.9184	-3.50561	-2.12374	-0.86117
N	1.37389	27.63018	0.331123	0.678577	-0.47968	-0.33642
N	1.344388	31.2882	-0.11488	2.933159	-0.26825	-0.10465
N	1.338051	32.63419	4.71248	1.553045	1.698634	-0.19623
O	1.373326	114.8392	-0.15437	-0.86083	-2.57693	-0.57849
C	1.41844	118.7037	-179.986	-1.61601	-3.77155	-0.69913
H	1.097084	111.5228	61.92237	-2.14823	-3.81624	-1.65743
H	1.090884	106.0802	-179.265	-0.89662	-4.5901	-0.64938
H	1.097058	111.5005	-60.3843	-2.33917	-3.87447	0.119401
H	2.088285	108.3171	-152.856	-3.09405	3.003293	-1.49171
C	2.453735	148.9837	154.007	-5.72009	-1.01833	-0.42652
H	1.105073	103.7227	152.8403	-5.75548	-1.79186	-1.21493
H	1.092727	82.40509	-101.363	-5.22552	-1.46529	0.439323
C	1.537225	140.9722	9.303333	-7.16676	-0.66563	-0.04468
H	1.093951	109.4508	86.2489	-7.71174	-0.34628	-0.93784
H	1.106322	109.3386	-30.3257	-7.16274	0.194989	0.6505
C	2.42292	140.8525	-176.492	-9.31537	-1.71427	0.348223
H	1.094321	90.08601	-90.121	-9.5686	-1.59562	-0.70976
H	1.094877	143.5262	31.15608	-9.79539	-2.62996	0.708566
H	1.107076	95.75137	161.7193	-9.75431	-0.86314	0.903661
C	2.435733	105.1339	-149.784	-5.42517	0.783587	-2.05083
H	1.087916	86.13505	14.32755	-4.82051	1.656518	-2.28736
H	1.10398	101.8421	122.6752	-5.3974	0.089837	-2.90915
H	1.092632	140.2714	-98.152	-6.4535	1.128801	-1.91971
N	1.425321	121.1802	178.4638	-4.93811	0.15478	-0.81914
N	1.458058	34.07505	37.22907	-7.87004	-1.82676	0.504175

Symbol	Bond	Angle	Dihedral	X	Y	Z
C	1.45841	110.5502	126.5604	-7.52412	-2.08702	1.896856
H	1.093164	110.7872	176.8763	-6.44576	-2.22685	2.009146
H	1.107253	112.9917	-62.0475	-7.83236	-1.27097	2.578819
H	1.0946	109.593	58.44441	-8.0116	-3.00868	2.230089
N	1.437829	117.84	-179.234	-3.23282	2.490068	-0.62681
O	2.268705	149.5674	-45.3702	-3.29105	4.5942	0.219515
C	1.226598	31.83767	-47.4283	-3.29984	3.385802	0.429848
C	1.491851	120.1415	-177.276	-3.444	2.868735	1.821782
H	1.086715	113.0442	-32.8426	-2.9861	3.515521	2.565387
C	1.33409	125.229	139.396	-4.18391	1.817878	2.179593
H	1.085609	121.4124	4.393163	-4.69447	1.207589	1.441068
H	1.086422	120.9142	-173.783	-4.3195	1.561231	3.226522
C	1.460822	147.7806	-1.33794	5.304837	-0.2245	0.154143
C	1.450252	130.9347	165.9303	6.672699	0.241304	0.030802
C	1.423405	106.4669	178.778	7.506511	-0.88528	0.279092
C	1.406445	135.5896	-3.5361	7.2933	1.460116	-0.29698
C	1.397599	122.9387	179.6671	8.902099	-0.82103	0.240511
C	1.39015	119.439	-178.865	8.681032	1.530771	-0.33852
H	1.083905	121.0071	-0.20377	6.70601	2.338553	-0.53841
C	1.390078	117.521	-0.52688	9.480399	0.405193	-0.06652
H	1.085619	121.4008	179.8058	9.514254	-1.69577	0.43712
H	1.085861	119.3362	-179.96	9.158436	2.472771	-0.59113
H	1.085691	119.5689	179.9575	10.56186	0.492901	-0.1049
C	1.381286	122.941	-14.7809	5.38351	-1.57189	0.447916
H	1.079665	127.9083	0.561794	4.570923	-2.26359	0.612008
N	1.369019	110.8375	-178.696	6.688642	-1.97634	0.533117
C	1.449345	126.1232	179.1129	7.149826	-3.31504	0.842654
H	1.095284	110.8756	120.026	7.753327	-3.71924	0.022864
H	1.095294	110.9175	-119.144	7.752812	-3.31925	1.757017
H	1.090825	108.9996	0.467848	6.285041	-3.963	0.991594
